# All-optical doubly resonant cavities for energy-efficient ReLU function in nanophotonic deep learning

**DOI:** 10.1371/journal.pone.0345850

**Published:** 2026-06-17

**Authors:** Amirreza Ahmadnejad, Mohammad Mehrdad Asadi, Somayyeh Koohi

**Affiliations:** 1 Department of Electrical Engineering, Sharif University of Technology, Tehran, Iran; 2 Department of Computer Engineering, Sharif University of Technology, Tehran, Iran; Oregon State University College of Engineering, UNITED STATES OF AMERICA

## Abstract

This paper presents a novel approach to implementing all-optical Rectified Linear Unit (ReLU) activation functions using compact doubly-resonant cavities with dimensions of approximately 10μm. The proposed design leverages $\chi^{\left(2\right)}$ nonlinear processes within carefully engineered photonic structures that simultaneously resonate at both fundamental and second-harmonic frequencies. By exploiting the phase-sensitive nature of second-harmonic generation, we demonstrate an optical analog to the ReLU function, achieving femtojoule-level activation energy—comparable to state-of-the-art approaches—while reducing device footprint by two orders of magnitude compared to previous implementations. The theoretical framework is developed using coupled-mode theory and validated through rigorous finite-difference time-domain simulations. Beyond ReLU, we show that the same physical structure can implement alternative activation functions such as ELU and GELU through simple adjustments to input conditions. Neural network simulations demonstrate that the proposed optical activation functions achieve classification accuracy within 0.4% of ideal electronic implementations while offering significant advantages in energy efficiency and processing speed. This work represents a significant advancement toward realizing energy-efficient, high-density optical neural networks for next-generation artificial intelligence hardware.

## 1. Introduction

The rapid advancement of deep learning and neural networks has led to unprecedented success in areas such as image recognition, audio processing [1], natural language processing, and autonomous systems [2,3]. However, the exponential growth in computational demands has outpaced improvements in traditional electronic computing hardware, particularly regarding energy efficiency [4]. Recent studies have quantified the substantial energy demands of large AI models, with Patterson et al. [5] estimating that training a large transformer model can consume over 1,000 MWh and produce hundreds of tons of CO_2_ equivalent emissions. Similar efficiency challenges exist in inference, where energy costs scale linearly with model size [6,7]. This energy bottleneck has prompted a search for alternative computing paradigms, with optical neural networks (ONNs) emerging as a promising solution due to their inherent parallelism and potential for high-speed, low-power operation [8,9,10,11]. Pioneering work by Miller [12] established the theoretical foundations for optical neural networks using meshes of Mach-Zehnder interferometers, while more recent reviews by Shastri et al. [9] and Miscuglio and Sorger [13] have highlighted the diverse approaches to photonic neural networks, from free-space diffractive implementations to integrated photonic circuits.

Optical implementations of neural networks can perform matrix multiplications and convolutions with remarkable efficiency by leveraging the wave nature of light [14,15]. These linear operations constitute the majority of computations in deep neural networks. However, the implementation of nonlinear activation functions—essential components that enable neural networks to model complex, non-linear relationships in data—presents significant challenges in the optical domain [8,16].

Among various activation functions, the Rectified Linear Unit (ReLU), defined as ReLU(x)=max(0,x), has become ubiquitous in modern deep learning architectures due to its favorable properties for gradient-based optimization and computational simplicity [17]. While electronic implementations of ReLU are straightforward, creating energy-efficient optical counterparts has proven challenging. Current approaches broadly fall into three categories: (1) optoelectronic conversion, which introduces latency and energy penalties [18]; (2) inherently nonlinear optical phenomena such as saturable absorption, which suffer from limited configurability [19]; and (3) nonlinear wave mixing, which typically requires high optical powers or long interaction lengths [20]. Beyond these approaches, researchers have explored various novel implementations of optical nonlinearities for neural networks, including wavelength-multiplexed resonant activation circuits [21], phase-change materials for programmable nonlinear functions [22], epsilon-near-zero materials for enhanced nonlinearities [23], and metasurface-based nonlinearities with subwavelength dimensions [24,25].

Recent work by Li et al. [20] demonstrated an all-optical ReLU function using periodically poled lithium niobate (PPLN) waveguides. Their approach leverages $\chi^{\left(2\right)}$ nonlinear processes—specifically, second harmonic generation (SHG) and degenerate optical parametric amplification (DOPA)—to implement the ReLU function with femtojoule-level energies and femtosecond response times. However, their implementation requires relatively long waveguides (approximately 2 mm) to achieve sufficient nonlinear interaction, primarily due to phase-matching or quasi-phase-matching (QPM) requirements. This presents a challenge for dense integration in photonic neural network architectures.

**Fig 1:**
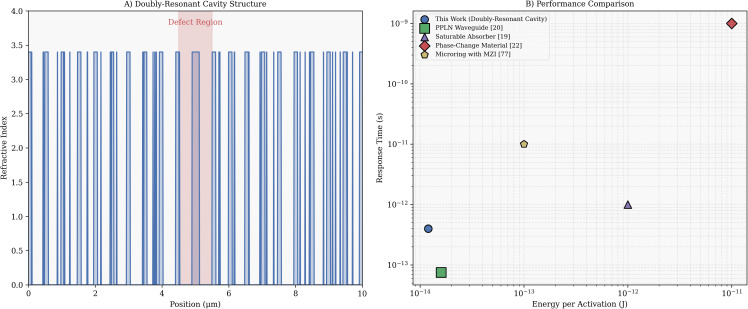
(A) Doubly-Resonant Cavity Structure showing the refractive index profile of alternating AlGaAs (n = 3.4) and air (n = 1.0) layers with a central defect region. The carefully engineered structure simultaneously resonates at both fundamental ($\omega_{1}$) and second-harmonic ($\omega_{2}=2\omega_{1}$) frequencies. **(B)** Performance comparison of our approach against existing optical activation technologies, showing the relationship between energy per activation and response time. Our doubly-resonant cavity approach achieves femtojoule-level activation energy with sub-picosecond response time while reducing device footprint by two orders of magnitude compared to previous implementations.

This paper introduces a novel method for implementing an all-optical ReLU function utilizing doubly-resonant cavities with dimensions of approximately 10 μm, significantly smaller—by about two orders of magnitude—than previously demonstrated devices. The underlying principle leverages theoretical insights from Rodriguez et al. [26], who established that perfect frequency conversion could occur at critical input powers within doubly-resonant nonlinear cavities. Through meticulous cavity design that ensures resonant modes at both fundamental frequency ω and its second harmonic 2ω, substantial field enhancement is achieved. This effectively reduces both the required interaction length and input power essential for efficient nonlinear optical operations. The key innovation of our approach is illustrated in Fig 1. We propose a compact doubly-resonant cavity with dimensions of approximately 10 μm that efficiently implements the ReLU function through nonlinear optical processes. The structure consists of alternating layers of AlGaAs and air, with a carefully designed defect region that enables simultaneous resonance at both fundamental and second-harmonic frequencies. By leveraging the phase-sensitive nature of $\chi^{\left(2\right)}$ nonlinear processes and the field enhancement provided by the resonant structure, we achieve both size reduction and energy efficiency compared to previous approaches. As shown in Fig 1(B), our device operates with femtojoule-level activation energy and sub-picosecond response time, offering significant advantages for integrated optical neural networks.

The study establishes a comprehensive theoretical framework for designing compact cavities suitable for optical ReLU functions by exploiting $\chi^{\left(2\right)}$ nonlinear processes. To complement this theoretical development, an analytical model based on coupled-mode theory is presented, enabling precise predictions of optical ReLU behavior. Numerical validation further supports the robustness of this model through detailed analytical simulations and finite-difference time-domain (FDTD) analyses. Additionally, the device showcases versatility, capable of implementing various activation functions such as ELU and GELU through simple adjustments of input power. Finally, the performance of the proposed optical component is evaluated within optical neural networks, emphasizing its potential effectiveness in image classification tasks.

The remainder of this paper is organized as follows: “Theoretical Framework” presents the theoretical framework for our approach, including coupled-mode equations and analytical solutions. “Design Methodology” details the design and optimization process for the doubly-resonant cavity structure. “Numerical Simulation Methods” describes our numerical simulation methodology and the results of our device characterization and comparison with ideal activation functions. In addition, we demonstrates the integration of our optical ReLU into a neural network for image classification. In “Discussion and Practical Implementation” discusses practical implementation considerations, and “Conclusion” concludes with a summary of our findings and directions for future research. All simulation codes, optimization algorithms, and analysis scripts used in this work are publicly available in our GitHub repository [27].

## 2. Theoretical framework

We establish the theoretical foundation for implementing an all-optical ReLU function using doubly-resonant optical cavities with $\chi^{\left(2\right)}$ nonlinearity. We develop the coupled-mode equations governing the system dynamics, derive analytical expressions predicting ReLU-like behavior, and analyze how the phase-sensitive nature of nonlinear optical interactions enables the implementation of the activation function.

Before developing the mathematical formalism, it is instructive to understand intuitively why doubly-resonant cavities can achieve efficient nonlinear frequency conversion in much smaller footprints compared to conventional phase-matched approaches. In traditional waveguide-based nonlinear optics, phase-matching is required to maintain the proper phase relationship between interacting waves over extended propagation distances, allowing the nonlinear effect to accumulate constructively. This typically requires millimeter-scale interaction lengths. In contrast, doubly-resonant cavities circumvent this length requirement through two key mechanisms. First, resonant enhancement significantly increases the field intensity within the cavity, effectively amplifying the nonlinear interaction strength. For a cavity with quality factor *Q*, the intracavity power is enhanced by a factor proportional to *Q* compared to the input power. Second, the frequency-matching condition ($\omega_{2}=2\omega_{1}$) replaces the phase-matching requirement, as both frequencies are simultaneously resonant within the same spatial structure. The cavity essentially “recycles” the light through the nonlinear medium many times, with each pass contributing to the nonlinear process in a phase-coherent manner. This allows the nonlinear interaction to build up over multiple cavity lifetimes rather than requiring long single-pass propagation, enabling miniaturization by approximately two orders of magnitude compared to conventional approaches.

**Fig 2:**
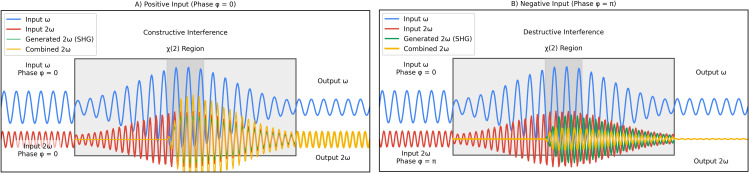
Operating principle of all-optical ReLU function using phase-sensitive nonlinear processes in a doubly-resonant cavity. **(A)** For positive inputs (phase ϕ=0), constructive interference between resonant 2ω and SHG-generated 2ω within the $\chi^{\left(2\right)}$ region results in an amplified second-harmonic output. **(B)** For negative inputs (phase ϕ=π), destructive interference suppresses the second-harmonic output due to phase cancellation.

The ReLU function, defined as ReLU(x)=max(0,x), is fundamental in modern deep neural networks. It passes positive inputs unchanged while setting negative inputs to zero, introducing essential nonlinearity that enables neural networks to approximate complex functions. To implement this behavior optically, we establish a mapping between the input signal and the optical output that reproduces this nonlinear transfer function. Fig 2 illustrates the operating principle of our proposed all-optical ReLU implementation. The doubly-resonant cavity simultaneously supports modes at both fundamental frequency $\omega_{1}$ and second-harmonic frequency $\omega_{2}=2\omega_{1}$. As shown in Fig 2(A), when a signal with phase $\phi_{1}=0$ (representing a positive input) enters the cavity, the SHG-generated field interferes constructively with the resonant field at $\omega_{2}$, resulting in an amplified output at the second-harmonic frequency. Conversely, Fig 2(B) demonstrates that when the input signal has phase $\phi_{1}=\pi$ (representing a negative input), destructive interference occurs between the SHG-generated field and the resonant field, suppressing the second-harmonic output.

Our approach encodes the input signal’s magnitude in the amplitude of the input field at fundamental frequency $\omega_{1}$, while its sign is encoded in the phase. Specifically, positive values correspond to a phase $\phi_{1}=0$, and negative values correspond to $\phi_{1}=\pi$. The output of the ReLU function is represented by the power of the second-harmonic field at frequency $\omega_{2}=2\omega_{1}$. The key physical insight underlying our implementation is that second-harmonic generation is inherently phase-sensitive. The efficiency of the SHG process depends on the phase relationship between the interacting fields, which allows us to selectively enhance or suppress frequency conversion based on the phase of the input signal, thereby implementing the rectification property of the ReLU function. Our approach leverages a photonic structure that simultaneously supports resonant modes at both fundamental frequency $\omega_{1}$ and second-harmonic frequency $\omega_{2}=2\omega_{1}$. In a linear regime, each cavity mode can be characterized by its resonant frequency $\omega_{k}$ and quality factor $Q_{k}=\omega_{k}\tau_{k}/2$, where $\tau_{k}$ is the cavity lifetime of mode *k*. Following the approach developed by Rodriguez et al. [26], we employ temporal coupled-mode theory to describe the field amplitudes *a*_*k*_(*t*) in each mode, normalized such that $|a_{k}{|}^{2}$ represents the electromagnetic energy stored in the mode.

For a passive cavity coupled to input/output channels, the linear dynamics are governed by:


$$\begin{array}{c} \frac{da_{k}}{dt}=\left(i\omega_{k}-\frac{1}{\tau_{k}}\right)a_{k}+\sqrt{\frac{2}{\tau_{s,k}}}s_{k+} \end{array}$$ (1)

where $s_{k+}$ is the amplitude of the incoming wave at frequency $\omega_{k}$, normalized such that $|s_{k+}{|}^{2}$ represents input power. The decay rate $1/\tau_{k}$ can be decomposed into $1/\tau_{k}=1/\tau_{e,k}+1/\tau_{s,k}$, where $1/\tau_{e,k}$ accounts for intrinsic losses (absorption, scattering) and $1/\tau_{s,k}$ represents coupling to the input/output channels [28]. The corresponding outgoing wave amplitude $s_{k-}$ is related to the cavity field by:


$$\begin{array}{c} s_{k-}=-s_{k+}+\sqrt{\frac{2}{\tau_{s,k}}}a_{k} \end{array}$$ (2)

For a doubly-resonant cavity, the structure must be carefully designed to ensure that $\omega_{2}=2\omega_{1}$ within a tolerance determined by the cavity linewidths $\Delta \omega_{k}=\omega_{k}/Q_{k}$. This frequency-matching condition is essential for efficient nonlinear interaction between the modes. When $\chi^{\left(2\right)}$ nonlinear material is introduced into the cavity, the fundamental and second-harmonic modes become coupled through nonlinear polarization. In a $\chi^{\left(2\right)}$ medium, the second-order nonlinear polarization is given by:


$$\begin{array}{c} P_{i}^{\left(2\right)}=\varepsilon_{0}\sum_{jk}\chi_{ijk}^{\left(2\right)}E_{j}E_{k} \end{array}$$ (3)

where $\chi_{ijk}^{\left(2\right)}$ is the second-order nonlinear susceptibility tensor, and *E*_*j*_ and *E*_*k*_ are the components of the electric field. The nonlinear polarization at frequency $\omega_{2}$ induced by the fundamental field is $P_{\omega_{2}}^{NL}=\varepsilon_{0}\chi^{\left(2\right)}E_{\omega_{1}}^{2}$, while the nonlinear polarization at frequency $\omega_{1}$ induced by the interaction of fundamental and second-harmonic fields is $P_{\omega_{1}}^{NL}=2\varepsilon_{0}\chi^{\left(2\right)}E_{\omega_{1}}^{*}E_{\omega_{2}}$ [29].

Using perturbation theory, we derive the coupling coefficients between the modes from the spatial overlap of the mode fields with the nonlinear polarization [26]. For a $\chi^{\left(2\right)}$ medium, these coupling coefficients are:


$$\begin{array}{c} \beta_{1}=\frac{1}{4}\frac{\int d^{3}x\sum_{ijk}\varepsilon \chi_{ijk}^{\left(2\right)}\left[E_{1i}^{*}(E_{2j}E_{1k}^{*}+E_{1j}^{*}E_{2k})\right]}{\left[\int d^{3}x\varepsilon |E_{1}{|}^{2}\right]{\left[\int d^{3}x\varepsilon |E_{2}{|}^{2}\right]}^{1/2}} \end{array}$$ (4)

$$\begin{array}{c} \beta_{2}=\frac{1}{4}\frac{\int d^{3}x\sum_{ijk}\varepsilon \chi_{ijk}^{\left(2\right)}E_{2i}^{*}E_{1j}E_{1k}}{\left[\int d^{3}x\varepsilon |E_{1}{|}^{2}\right]{\left[\int d^{3}x\varepsilon |E_{2}{|}^{2}\right]}^{1/2}} \end{array}$$ (5)

where *E*_1_ and *E*_2_ are the normalized spatial mode profiles at frequencies $\omega_{1}$ and $\omega_{2}$, respectively. For energy conservation, these coefficients must satisfy $\omega_{1}\beta_{1}=\omega_{2}\beta_{2}^{*}$ [26]. The detailed derivation of these coupling coefficients is provided in S1 in S1 Appendix.

The magnitude of these coupling coefficients directly impacts the efficiency of the nonlinear interaction. For a given $\chi^{\left(2\right)}$ material, the coupling strength is maximized when the spatial overlap between the modes is high in regions with nonlinear material. This underscores the importance of careful mode engineering in the cavity design process. Incorporating these nonlinear coupling terms into the temporal coupled-mode theory, we obtain the following coupled-mode equations for the fundamental and second-harmonic fields:


$$\begin{array}{c} \frac{da_{1}}{dt}=\left(i\omega_{1}-\frac{1}{\tau_{1}}\right)a_{1}-i\omega_{1}\beta_{1}a_{1}^{*}a_{2}+\sqrt{\frac{2}{\tau_{s,1}}}s_{1+} \end{array}$$ (6)

$$\begin{array}{c} \frac{da_{2}}{dt}=\left(i\omega_{2}-\frac{1}{\tau_{2}}\right)a_{2}-i\omega_{2}\beta_{2}a_{1}^{2}+\sqrt{\frac{2}{\tau_{s,2}}}s_{2+} \end{array}$$ (7)

where *a*_1_ and *a*_2_ are the complex amplitudes of the fundamental and second-harmonic modes, respectively. The terms $-i\omega_{1}\beta_{1}a_{1}^{*}a_{2}$ and $-i\omega_{2}\beta_{2}a_{1}^{2}$ represent the nonlinear coupling due to the $\chi^{\left(2\right)}$ interaction. The input fields *s*_1+_ and *s*_2+_ drive the system at frequencies $\omega_{1}$ and $\omega_{2}$, respectively [30]. These coupled-mode equations capture the essential physics of the nonlinear interaction in the cavity. The term $-i\omega_{2}\beta_{2}a_{1}^{2}$ in Equation 7 describes the second harmonic generation process, where two photons at frequency $\omega_{1}$ combine to produce a photon at frequency $\omega_{2}$. Conversely, the term $-i\omega_{1}\beta_{1}a_{1}^{*}a_{2}$ in Equation 6 describes the reverse process of degenerate optical parametric amplification (DOPA), where a photon at frequency $\omega_{2}$ splits into two photons at frequency $\omega_{1}$. These equations are derived under the rotating wave approximation and assume that the nonlinearity is weak enough to be treated perturbatively. These coupled-mode equations follow the formalism developed by Haus [28] and extended by Suh et al. [31] for nonlinear optical resonators. This approach has become standard for analyzing resonant nonlinear optical systems [32] and has been successfully applied to a wide range of photonic devices, from optical modulators to frequency combs [33].

For the implementation of an optical ReLU function, we are primarily interested in the steady-state behavior of the cavity. In the steady state, $da_{1}/dt=da_{2}/dt=0$, and we can solve Equations 6 and 7 for the field amplitudes. Assuming operation near resonance, we can simplify the analysis by setting $\omega_{1}\approx \omega_{1,\text{res}}$ and $\omega_{2}\approx \omega_{2,\text{res}}$, where $\omega_{k,\text{res}}$ are the resonant frequencies of the cavity. From Equations 6 and 7, we obtain:


$$\begin{array}{c} a_{1}=\frac{\sqrt{\frac{2}{\tau_{s,1}}}s_{1+}-i\omega_{1}\beta_{1}a_{1}^{*}a_{2}}{\frac{1}{\tau_{1}}-i\Delta \omega_{1}} \end{array}$$ (8)

$$\begin{array}{c} a_{2}=\frac{\sqrt{\frac{2}{\tau_{s,2}}}s_{2+}-i\omega_{2}\beta_{2}a_{1}^{2}}{\frac{1}{\tau_{2}}-i\Delta \omega_{2}} \end{array}$$ (9)

where $\Delta \omega_{k}=\omega_{k}-\omega_{k,\text{res}}$ represents the detuning from resonance. For perfect frequency conversion, where all power at the fundamental frequency is converted to the second harmonic, we require $s_{1-}=0$. Using Equation 2, this implies:


$$\begin{array}{c} s_{1-}=-s_{1+}+\sqrt{\frac{2}{\tau_{s,1}}}a_{1}=0 \end{array}$$ (10)

Following the approach in [26], and assuming operation exactly at resonance ($\Delta \omega_{k}=0$), we derive the critical input power for 100% frequency conversion:


$$\begin{array}{c} P_{\text{critical}}=|s_{1+}{|}^{2}=\frac{\omega_{1}}{2|\beta_{1}{|}^{2}Q_{1}Q_{2}} \end{array}$$ (11)

where $Q_{1}=\omega_{1}\tau_{1}/2$ and $Q_{2}=\omega_{2}\tau_{2}/2$ are the quality factors of the fundamental and second-harmonic modes, respectively. The detailed derivation of this critical power is presented in S1 in S1 Appendix. The concept of doubly-resonant nonlinear cavities has been explored theoretically by Berger [34] and experimentally demonstrated by Bruch et al. [35] for efficient second-harmonic generation. Further theoretical developments by Burgess et al. [36] and Lin et al. [37] have extended this framework to various $\chi^{\left(2\right)}$ processes, establishing the fundamental limits and optimization strategies for such systems. Recent experimental work by Lu et al. [38] has demonstrated record-high conversion efficiencies in lithium niobate microrings, confirming the advantages of the doubly-resonant approach.

This expression reveals a crucial insight: the critical power scales inversely with the product of the quality factors and the square of the nonlinear coupling coefficient. By designing a cavity with high *Q*-factors and strong nonlinear coupling, we can achieve efficient frequency conversion at very low input powers, which is essential for energy-efficient optical neural networks.

To implement a ReLU function, we establish a mapping between the input signal and the output that approximates ReLU(x)=max(0,x). In our approach, we encode the input signal’s magnitude in the amplitude of the input field at frequency $\omega_{1}$, while its sign is encoded in the phase. Specifically, positive values correspond to $\phi_{1}=0$, and negative values correspond to $\phi_{1}=\pi$. By setting *s*_2+_ = 0 (no external input at $\omega_{2}$) and using a fixed bias input at $\omega_{1}$, we analyze the cavity’s response. At steady state, the output at $\omega_{2}$ is:


$$\begin{array}{c} s_{2-}=\sqrt{\frac{2}{\tau_{s,2}}}a_{2} \end{array}$$ (12)

The phase-sensitive nature of SHG means that the conversion efficiency depends on the relative phase between the interacting fields. For positive inputs (where $\phi_{1}=0$), efficient SHG occurs, producing an output at $\omega_{2}$. For negative inputs (where $\phi_{1}=\pi$), the SHG process is effectively suppressed due to destructive interference. This phase-dependent behavior forms the basis of our ReLU implementation.

To understand this more quantitatively, consider the steady-state solution for *a*_2_ when *s*_2+_ = 0:


$$\begin{array}{c} a_{2}=\frac{-i\omega_{2}\beta_{2}a_{1}^{2}}{\frac{1}{\tau_{2}}-i\Delta \omega_{2}} \end{array}$$ (13)

The squared amplitude $a_{1}^{2}$ depends on the phase of the input field *s*_1+_. For $\phi_{1}=0$, $a_{1}^{2}$ has a positive phase, leading to efficient generation of *a*_2_. For $\phi_{1}=\pi$, $a_{1}^{2}$ has a negative phase, resulting in destructive interference that suppresses the generation of *a*_2_. By calibrating the input-output relationship, we achieve a transfer function that closely approximates the ReLU function:


$$\begin{array}{c} |s_{2-}{|}^{2}\approx \left\{ \begin{array}{cc} \alpha |s_{1+}{|}^{2}, & \text{if }\phi_{1}=0\text{ (positive input)}\\ 0, & \text{if }\phi_{1}=\pi \text{ (negative input)} \end{array}\right. \end{array}$$ (14)

where α is a scaling factor determined by the cavity parameters and operating conditions. The exact form of the transfer function depends on the detailed parameters of the cavity and the operating conditions. Through numerical solution of the coupled-mode equations, we obtain more precise predictions of the device behavior, as discussed in Numerical Simulation Methods. This phase-sensitive approach to optical nonlinearity builds upon established techniques in nonlinear optics. Similar phase-dependent effects have been exploited in phase-sensitive amplifiers [39], optical parametric oscillators [40], and quantum optics [41]. Recent work by Bosshard et al. [42] demonstrated precise phase control in nanophotonic $\chi^{\left(2\right)}$ devices, while Zhang et al. [43] leveraged phase sensitivity for optical switching applications. This phase-dependent behavior is crucial for our ReLU implementation, as it enables the selective suppression or enhancement of frequency conversion based on the input phase.

The phase-encoding scheme employed in our implementation is naturally compatible with coherent optical neural network architectures where phase information is preserved throughout computation. In our approach, the sign of the input signal is encoded in the optical phase ($\phi_{1}=0$ for positive inputs, $\phi_{1}=\pi$ for negative inputs), while the magnitude is encoded in the field amplitude. This encoding aligns directly with interferometric approaches based on Mach-Zehnder interferometer (MZI) meshes [14,44], which are among the most widely studied and scalable architectures for optical matrix multiplication.

In MZI-based systems, the output fields inherently carry both amplitude and phase information. The linear operations performed by the MZI mesh produce outputs where constructive and destructive interference naturally generate phase relationships of 0 or π relative to a reference, depending on whether the effective matrix element is positive or negative [45]. Consequently, the transition from the MZI-based linear layer to our phase-sensitive ReLU is seamless, requiring no additional phase encoding circuitry between the linear and nonlinear optical components.

Similar phase-based encoding schemes have been successfully demonstrated in compact nanophotonic computing architectures. Qu et al. [46] employed phase-coherent optical scattering units for neural network operations, achieving high-precision matrix multiplications in $4\times 4\;\mu {\text{m}}^{2}$ footprints. The phase-sensitive nature of $\chi^{\left(2\right)}$ processes has been extensively utilized in optical signal processing applications [42,43], confirming the robustness and practicality of phase-dependent optical operations in integrated photonic systems.

For integration with incoherent or intensity-based optical computing approaches, such as those employing wavelength-division multiplexing or free-space diffractive systems [47], a phase modulator can be inserted before our ReLU unit to convert the sign information into the appropriate phase encoding. Modern integrated electro-optic phase modulators in thin-film lithium niobate operate at speeds exceeding 100 GHz with insertion losses below 1 dB [48], adding minimal overhead to the overall system while enabling compatibility with a broader range of optical neural network architectures.

Our framework can be extended to implement other activation functions by adjusting the input conditions. For example, by introducing a small external field at $\omega_{2}$ with appropriate phase, we can implement functions similar to ELU (Exponential Linear Unit) or GELU (Gaussian Error Linear Unit) [49,50]. For an ELU-like function with parameter α:


$$\begin{array}{c} \text{ELU}(x)=\left\{ \begin{array}{cc} x, & \text{if }x>0\\ \alpha (e^{x}-1), & \text{if }x\leq 0 \end{array}\right. \end{array}$$ (15)

This can be approximated by setting $s_{2+}=Ae^{i\phi_{2}}$ with appropriate amplitude *A* and phase $\phi_{2}$. The external field *s*_2+_ interferes with the generated second-harmonic field, creating a nonlinear response for negative inputs that resembles the exponential term in the ELU function. Similarly, for GELU and other activation functions, different bias conditions and phase relationships can be employed. The detailed implementation of these alternative activation functions is presented in Numerical Simulation Methods, along with numerical validation.

Several factors determine the performance of our optical ReLU implementation. First, the approximation of the ideal ReLU function is influenced by the cavity parameters and operating conditions. The quality of the approximation can be quantified by metrics such as the coefficient of determination (*R*^2^) or mean squared error (MSE) between the device response and the ideal ReLU function. Second, the energy efficiency, characterized by the energy per activation, is fundamentally limited by the critical power derived in Equation 11. This depends on the cavity design through the quality factors *Q*_1_ and *Q*_2_, and the nonlinear coupling coefficient $\beta_{1}$. Third, the response time of the device is limited by the cavity lifetime $\tau_{k}=2Q_{k}/\omega_{k}$. Higher quality factors, while reducing the critical power, increase the response time, creating a trade-off between energy efficiency and speed. Fourth, the dynamic range of the device is limited by the validity of the perturbative approach used to derive the coupled-mode equations. At very high input powers, higher-order nonlinear effects may become significant, causing deviations from the predicted behavior.

## 3. Design methodology

This section presents a systematic methodology for designing a compact doubly-resonant cavity that effectively implements the all-optical ReLU function. We begin with general design requirements, proceed through the linear cavity design, nonlinear mode coupling optimization, and input/output coupling design, culminating in a fully optimized structure.

The effective implementation of an all-optical ReLU function using doubly-resonant cavities requires meeting several key design criteria simultaneously. First, the structure must support optical modes at both the fundamental frequency $\omega_{1}$ and second-harmonic frequency $\omega_{2}=2\omega_{1}$, with precise frequency matching to within the resonance linewidths. Second, both resonances should exhibit high quality factors to enhance the nonlinear interaction while maintaining reasonable bandwidth. Third, the mode profiles must have strong spatial overlap in regions with $\chi^{\left(2\right)}$ nonlinearity to maximize the coupling coefficients. Fourth, the cavity must be efficiently coupled to input/output channels for practical operation.

These requirements guide our multi-objective optimization framework, which can be formulated as maximizing a figure of merit:


$$\begin{array}{c} F=w_{1}Q_{1}+w_{2}Q_{2}+w_{3}|\beta |-w_{4}\Delta_{\omega }-w_{5}E_{transfer} \end{array}$$ (16)

where *w*_*i*_ are weights for each objective, $\Delta_{\omega }=|\omega_{2}-2\omega_{1}|$ represents the frequency mismatch, and *E*_*transfer*_ is the error in the transfer function compared to the ideal ReLU function. The weights are chosen to balance competing objectives based on their relative importance for our application. Our multi-objective optimization approach builds on established methodologies for photonic crystal design, similar to those developed by Piggott et al. [51] for inverse design of photonic devices and Lu et al. [52] for high-Q resonator optimization. We incorporate elements from robust optimization techniques [53] to ensure design tolerance to fabrication variations, while utilizing efficient global search algorithms inspired by Molesky et al. [54] for photonic structure optimization.

Our design workflow proceeds through several integrated stages: linear cavity design for double resonance, quality factor engineering, nonlinear mode coupling optimization, input/output coupling design, and full device characterization and refinement. For each stage, we employ a combination of analytical methods and numerical simulations, with the transfer matrix method serving as the primary analytical tool for the linear design and the coupled-mode equations for nonlinear performance evaluation.

### 3.1. Linear cavity design

We begin with a quarter-wave stack structure, which provides a natural framework for creating optical resonances with controlled frequencies. For a target fundamental wavelength $\lambda_{1}$ (corresponding to frequency $\omega_{1}=2\pi c/\lambda_{1}$), the layer thicknesses in a quarter-wave stack are given by [55]:


$$\begin{array}{c} d_{1}=\frac{\lambda_{1}}{4n_{1}},\;d_{2}=\frac{\lambda_{1}}{4n_{2}} \end{array}$$ (17)

where *n*_1_ and *n*_2_ are the refractive indices of the alternating layers.

The complete structural configuration of our doubly-resonant cavity is presented in Table 1. The cavity consists of a one-dimensional photonic crystal structure with alternating high- and low-refractive-index layers forming distributed Bragg reflector (DBR) mirrors on both sides of a central defect region. The high-index layers are aluminum gallium arsenide (Al${\;}_{0.3}{\text{Ga}}_{0.7}$As) with refractive index $n_{H}=3.40$ at $\lambda_{1}=1550$ nm, while the low-index layers are air gaps ($n_{L}=1.00$) created through undercut etching of a sacrificial layer. The quarter-wave layer thicknesses are $d_{H}=\lambda_{1}/(4n_{H})=114$ nm for the high-index layers and $d_{L}=\lambda_{1}/(4n_{L})=388$ nm for the low-index layers, resulting in a lattice period of $\Lambda =d_{H}+d_{L}=502$ nm.

**Table 1:** Complete structural parameters of the doubly-resonant cavity.

Parameter	Description	Value
*Material Properties*		
*n*_*H*_	High-index (AlGaAs) refractive index	3.40
*n*_*L*_	Low-index (air) refractive index	1.00
$\chi^{\left(2\right)}$	Second-order nonlinear susceptibility	100 pm/V
*d*_eff_	Effective nonlinear coefficient	50 pm/V
*Quarter-Wave Stack (DBR Mirrors)*		
*d*_*H*_	High-index layer thickness	114 nm
*d*_*L*_	Low-index layer thickness	388 nm
Λ	Lattice period (unit cell)	502 nm
*N*_*L*_	Number of layer pairs per mirror	7
*L*_*DBR*_	Total DBR mirror length (one side)	3.51 μm
*Defect Region*		
$d_{H^{\prime }}$	Outer modified high-index layers	142 nm
$d_{H^{\prime \prime }}$	Center modified high-index layer	165 nm
$d_{L,\text{defect}}$	Low-index layers in defect	388 nm
*L*_defect_	Total defect region length	1.23 μm
*Transverse Waveguide Dimensions*		
*w*	Ridge waveguide width	1.8 μm
*h*_total_	Total AlGaAs film thickness	700 nm
*h*_ridge_	Ridge etch depth	400 nm
*A*_eff_	Effective mode area	2.1 $\mu {\text{m}}^{2}$
*Overall Cavity*		
*L*_total_	Total cavity length	8.25 μm
–	Device footprint	10 × 2 $\mu {\text{m}}^{2}$
*Design Wavelengths*		
$\lambda_{1}$	Fundamental wavelength	1550 nm
$\lambda_{2}$	Second-harmonic wavelength	775 nm
$\omega_{1}$	Fundamental frequency	193.55 THz
$\omega_{2}$	Second-harmonic frequency	387.10 THz

Each DBR mirror comprises $N_{L}=7$ quarter-wave layer pairs, providing sufficient reflectivity while maintaining a compact overall device length. The central defect region is engineered to support simultaneous resonances at both the fundamental frequency $\omega_{1}$ and second-harmonic frequency $\omega_{2}=2\omega_{1}$. Following the design principles for doubly-resonant nonlinear cavities [56], the defect consists of a modified five-layer structure (H^′^-L-H^″^-L-H^′^) with optimized layer thicknesses: the outer high-index layers have thickness $d_{H^{\prime }}=142$ nm and the central high-index layer has thickness $d_{H^{\prime \prime }}=165$ nm, while the low-index layers maintain the standard quarter-wave thickness $d_{L,\text{defect}}=388$ nm. These dimensions were obtained through our optimization procedure (described in “Input/Output Coupling and Final Optimization”) to simultaneously satisfy the resonance conditions at both $\omega_{1}$ and $\omega_{2}=2\omega_{1}$.

The total longitudinal extent of the cavity is *L*_total_ = 8.25 μm, comprising two DBR sections (2 × 3.51 μm) and the defect region (1.23 μm). The transverse waveguide dimensions are: ridge width *w* = 1.8 μm, total film thickness *h*_total_ = 700 nm, and ridge etch depth *h*_ridge_ = 400 nm. The effective mode area at the fundamental wavelength, calculated from the transverse mode profile overlap integral, is $A_{\text{eff}}\approx 2.1$ $\mu {\text{m}}^{2}$. Including input/output waveguide coupling regions, the complete device footprint is approximately 10 × 2 $\mu {\text{m}}^{2}$. The second-order nonlinear susceptibility of AlGaAs is $\chi^{\left(2\right)}\approx 100$ pm/V [57], with an effective nonlinear coefficient $d_{\text{eff}}\approx 50$ pm/V accounting for crystal orientation.

An important property of quarter-wave stacks is that they naturally create photonic band gaps at both $\omega_{1}$ and $2\omega_{1}$, making them ideal starting points for our design [58]. For our implementation, we select materials with high refractive index contrast to achieve wide photonic band gaps. We use AlGaAs (n≈3.4 at 1550 nm) and air (*n* = 1.0) for the high and low index layers, respectively. This material system offers several advantages for our application, including a strong second-order nonlinear susceptibility ($\chi^{\left(2\right)}\approx 100$ pm/V) in AlGaAs and well-established fabrication techniques for creating suspended membrane structures. The target wavelength is set to $\lambda_{1}=1550$ nm, a standard telecommunications wavelength that allows for compatibility with existing photonic integrated circuit platforms. To accurately model the electromagnetic response of the multilayer structure, we employ the transfer matrix method (TMM). For a single layer with refractive index *n* and thickness *d*, the transfer matrix for transverse electric (TE) polarization is [59]:


$$\begin{array}{c} M=(\begin{array}{cc} \cos(kd) & \frac{i}{p}\sin(kd)\\ ip\sin(kd) & \cos(kd) \end{array}) \end{array}$$ (18)

where $k=\frac{\omega }{c}n$ is the wave number in the medium, and *p* = *n* for TE polarization. For transverse magnetic (TM) polarization, *p* = 1/*n*. The total transfer matrix for a multilayer structure with *N* layers is calculated as the product of the individual layer matrices:


$$\begin{array}{c} M_{total}=M_{1}\cdot M_{2}\cdot ...\cdot M_{N} \end{array}$$ (19)

From the total transfer matrix, we can calculate the reflection and transmission coefficients:


$$\begin{array}{c} r=\frac{\left(M_{11}+M_{12}p_{0})p_{N}-(M_{21}+M_{22}p_{0}\right)}{\left(M_{11}+M_{12}p_{0})p_{N}+(M_{21}+M_{22}p_{0}\right)} \end{array}$$ (20)

$$\begin{array}{c} t=\frac{2p_{0}}{\left(M_{11}+M_{12}p_{0})p_{N}+(M_{21}+M_{22}p_{0}\right)} \end{array}$$ (21)

where *p*_0_ and *p*_*N*_ are the impedances of the incident and exit media, respectively. The reflection and transmission intensities are then *R* = |*r*|^2^ and $T=\frac{p_{N}}{p_{0}}|t{|}^{2}$.

Using the TMM, we can efficiently calculate the frequency response of multilayer structures and analyze the properties of resonant modes, including their central frequencies, quality factors, and field distributions.

To create a cavity that resonates at both $\omega_{1}$ and $\omega_{2}$, we introduce a defect in the periodic quarter-wave stack. For a conventional single-resonant cavity, a defect with optical thickness $\lambda_{1}/2$ creates a resonance at $\omega_{1}$. However, for double resonance, a more complex defect structure is required.

We implement a compound defect consisting of multiple layers with carefully optimized thicknesses. A common configuration is a three-layer defect with structure $H^{\prime }LH^{\prime }$, where $H^{\prime }$ represents a high-index layer with non-standard thickness. The thicknesses are optimized to satisfy:


$$\begin{array}{c} \omega_{1}\cdot L_{eff,1}=m_{1}\pi c,\;\omega_{2}\cdot L_{eff,2}=m_{2}\pi c \end{array}$$ (22)

where *L*_*eff*,1_ and *L*_*eff*,2_ are the effective optical path lengths for the fundamental and second-harmonic modes, respectively, and *m*_1_ and *m*_2_ are integers satisfying $m_{2}=2m_{1}$ [60]. For our design, we use a modified defect structure with five layers: $H^{\prime }LH^{\prime \prime }LH^{\prime }$, where $H^{\prime }$ and $H^{\prime \prime }$ are high-index layers with different non-standard thicknesses. This additional degree of freedom allows better control over the frequency matching between the two resonances. Achieving exact frequency matching ($\omega_{2}=2\omega_{1}$) requires careful fine-tuning of the structure. We employ numerical optimization to minimize the frequency mismatch $\Delta =|\omega_{2}-2\omega_{1}|$. The optimization parameters include defect layer thicknesses and, if necessary, slight adjustments to the thicknesses of surrounding layers. We use a simulated annealing algorithm to explore the parameter space efficiently and avoid local minima (see S4 in S1 Appendix for implementation details).

The dispersion properties of the cavity materials and structure play a critical role in achieving precise frequency matching between the fundamental and second-harmonic resonances. Material dispersion of AlGaAs is modeled using the Sellmeier equation [61]:


$$\begin{array}{c} n^{2}(\lambda )=A+\frac{B\lambda^{2}}{\lambda^{2}-C^{2}} \end{array}$$ (23)

where the parameters for Al${\;}_{0.3}{\text{Ga}}_{0.7}$As are *A* = 10.906, *B* = 0.97501 $\mu {\text{m}}^{2}$, and *C* = 0.52886 μm. At the fundamental wavelength $\lambda_{1}=1550$ nm, this yields $n(\lambda_{1})=3.40$, while at the second-harmonic wavelength $\lambda_{2}=775$ nm, we obtain $n(\lambda_{2})=3.52$. The material dispersion parameter, quantified by the wavelength derivative dn/dλ, is approximately −0.015 $\mu {\text{m}}^{-1}$ at $\lambda_{1}$ and −0.048 $\mu {\text{m}}^{-1}$ at $\lambda_{2}$. This dispersion must be carefully accounted for in the cavity design to ensure that the optical path lengths satisfy the resonance conditions at both wavelengths simultaneously.

The group index, which determines the velocity of optical pulses in the cavity, is given by $n_{g}=n-\lambda (dn/d\lambda )$. For our AlGaAs material at $\lambda_{1}=1550$ nm, we calculate $n_{g,1}\approx 3.42$, and at $\lambda_{2}=775$ nm, $n_{g,2}\approx 3.56$. The group velocity dispersion (GVD) parameter, defined as $\beta_{2}=(\lambda^{3}/2\pi c^{2})(d^{2}n/d\lambda^{2})$, is approximately $\beta_{2,1}\approx 180$ fs^2^/mm at the fundamental wavelength and $\beta_{2,2}\approx 420$ fs^2^/mm at the second harmonic. These moderate dispersion values ensure that femtosecond pulses experience minimal temporal broadening during propagation through the cavity, preserving the ultrafast response time critical for high-speed neural network operations.

The cavity dispersion is characterized by the free spectral range (FSR), which represents the frequency spacing between adjacent longitudinal modes. For our cavity with effective length $L_{\text{eff}}\approx 8.25$ μm and effective refractive index $n_{\text{eff}}\approx 2.4$ (accounting for the alternating high and low index layers), the FSR is:


$$\begin{array}{c} \text{FSR}=\frac{c}{2n_{\text{eff}}L_{\text{eff}}}\approx 7.6\text{ THz} \end{array}$$ (24)

This large FSR ensures that the fundamental and second-harmonic resonances are well-isolated from neighboring cavity modes, preventing unwanted mode coupling. The precision of frequency matching between $\omega_{2}$ and $2\omega_{1}$ is critical for efficient nonlinear conversion. In our optimized design, we achieve $|\omega_{2}-2\omega_{1}|/\omega_{1}<5.2\times 10^{-4}$, which is well within the cavity linewidths determined by the quality factors $Q_{1}=5.3\times 10^{3}$ and $Q_{2}=6.8\times 10^{3}$ (corresponding to $\Delta \omega_{1}/\omega_{1}=1.9\times 10^{-4}$ and $\Delta \omega_{2}/\omega_{2}=1.5\times 10^{-4}$).

The acceptance bandwidth of the nonlinear process, which determines the range of input wavelengths over which efficient frequency conversion occurs, is primarily limited by the cavity resonance linewidths rather than phase-matching considerations as in conventional waveguide approaches. The resonance linewidth at the fundamental frequency is $\Delta \lambda_{1}=\lambda_{1}^{2}/(2\pi cn_{g}Q_{1})\approx 0.29$ nm, corresponding to a bandwidth of approximately 36 GHz. This bandwidth is sufficient to accommodate femtosecond pulses while maintaining high conversion efficiency. Temperature tuning provides an additional degree of freedom for fine-tuning the resonance frequencies. The thermo-optic coefficient of AlGaAs is $dn/dT\approx 2\times 10^{-4}$ K^−1^ [61], resulting in a resonance frequency shift of approximately $d\omega_{1}/dT\approx -11$ GHz/K. This allows for precise adjustment of the frequency matching condition over a temperature range of a few degrees Celsius, compensating for fabrication variations and enabling optimal device performance.

Compared to conventional phase-matched waveguide approaches, our resonant cavity design offers distinct advantages in terms of dispersion management. In periodically-poled waveguides, achieving quasi-phase-matching requires precise control of the poling period over millimeter-scale interaction lengths, with tight fabrication tolerances. Moreover, the phase-matching bandwidth in such devices is fundamentally limited by the coherence length, typically resulting in acceptance bandwidths of a few nanometers. In contrast, our doubly-resonant cavity achieves frequency conversion within a compact 10 μm footprint, with the acceptance bandwidth determined by the cavity linewidth rather than phase-matching considerations. This relaxes fabrication tolerances while maintaining comparable bandwidth, as demonstrated by the negligible performance variation observed in our tolerance analysis (mention in “Input/Output Coupling and Final Optimization”).

### 3.2. Quality factor and nonlinear coupling optimization

The quality factors of the resonant modes directly impact the critical power for nonlinear conversion. From coupled-mode theory, the critical power is given by [26]:


$$\begin{array}{c} P_{critical}=|s_{1+}{|}^{2}=\frac{\omega_{1}}{2|\beta_{1}{|}^{2}Q_{1}Q_{2}} \end{array}$$ (25)

To achieve efficient conversion at low powers, we aim to maximize the product $Q_{1}Q_{2}$ while maintaining frequency matching. The quality factors are controlled by the number of quarter-wave layer pairs on each side of the defect, the refractive index contrast between high and low-index materials, and the coupling to input/output channels. For each resonant mode, the quality factor is calculated from the transmission spectrum using $Q_{k}=\frac{\omega_{k,res}}{\Delta \omega_{k}}$, where $\Delta \omega_{k}$ is the full width at half maximum of the transmission peak at resonance $\omega_{k,res}$.

In our design, we use a total of *N* = 7 quarter-wave pairs on each side of the defect to achieve high quality factors while maintaining a compact overall size. The theoretical quality factors for such a structure, assuming no intrinsic material losses, can be estimated by [58]:


$$\begin{array}{c} Q_{k}\approx \frac{\omega_{k,res}}{2c}\cdot \frac{(n_{H}/n_{L}{)}^{2N}-1}{(n_{H}/n_{L}{)}^{N}}L_{eff,k} \end{array}$$ (26)

where *n*_*H*_ and *n*_*L*_ are the high and low refractive indices, respectively, and *L*_*eff*,*k*_ is the effective cavity length for mode *k*. With our material selection and structural parameters, we target quality factors of $Q_{1}\approx 10^{4}$ and $Q_{2}\approx 10^{4}$, which are sufficient to achieve sub-femtojoule critical powers while maintaining practical bandwidth and fabrication tolerances.

After designing the linear structure, we calculate the electric field distributions *E*_1_(*z*) and *E*_2_(*z*) at the resonant frequencies $\omega_{1}$ and $\omega_{2}$. These field distributions determine the nonlinear coupling strength through the overlap integrals in the coupling coefficients. Using the transfer matrix method, we calculate the field at any position *z* within the structure:


$$\begin{array}{c} \left(\begin{array}{c} E(z)\\ H(z) \end{array})=M(0,z)(\begin{array}{c} 1+r\\ p_{0}(1-r) \end{array}\right) \end{array}$$ (27)

where *M*(0, *z*) is the transfer matrix from the input interface to position *z*, and *r* is the reflection coefficient. The fields are normalized according to $\int \varepsilon (z)|E_{1}(z){|}^{2}dz=1$ and $\int \varepsilon (z)|E_{2}(z){|}^{2}dz=1$. The nonlinear coupling coefficient is then calculated using:


$$\begin{array}{c} \beta =\frac{\omega_{1}}{4}\frac{\int \varepsilon (z)\chi^{\left(2\right)}(z)E_{1}^{2}(z)E_{2}^{*}(z)dz}{\sqrt{\int \varepsilon (z)|E_{1}(z){|}^{2}dz\cdot \int \varepsilon (z)|E_{2}(z){|}^{2}dz}} \end{array}$$ (28)

To maximize this coupling coefficient, we place the $\chi^{\left(2\right)}$ nonlinear material in regions where the fields *E*_1_(*z*) and *E*_2_(*z*) have strong spatial overlap [62]. In our design, we utilize AlGaAs throughout the structure, which has a strong $\chi^{\left(2\right)}$ nonlinearity ($\chi^{\left(2\right)}\approx 100$ pm/V) [57], with particular attention to the central defect region where the field intensity is maximum for both modes. Our material selection leverages this strong second-order nonlinearity of AlGaAs, which has been extensively characterized by Stegeman et al. [57] and utilized in integrated nonlinear devices by Kuo et al. [63]. For the central defect region, we carefully design the AlGaAs structure as demonstrated in similar work by Surya et al. [64], who have optimized the crystalline orientation for maximum nonlinear efficiency. Recent work by Bruch et al. [65] has demonstrated the integration of AlGaAs in high-Q resonators, confirming its suitability for our application.

We optimize the position and thickness of the AlN layer to maximize the coupling coefficient while maintaining the resonance conditions. For the final structure, we achieve a normalized coupling coefficient of $|\beta |\approx 10^{-3}$, which is sufficient for low-power ReLU operation.

### 3.3. Input/output coupling and final optimization

For practical implementation, the cavity must be coupled to input/output waveguides. We design the coupling strength to balance two key considerations. First, for optimal power transfer to the cavity, the external coupling rate should match the intrinsic loss rate (critical coupling condition). Second, the total quality factor affects the critical power for frequency conversion. The coupling is implemented by reducing the number of quarter-wave layers on the input/output side of the cavity, creating a partially transmitting mirror. The coupling strength is controlled by the number of layers and is designed to achieve $\frac{1}{\tau_{s,k}}\approx \frac{1}{\tau_{e,k}}$ for the fundamental mode, where $\tau_{s,k}$ is the external coupling lifetime and $\tau_{e,k}$ is the intrinsic lifetime due to losses.

In our final design, we use 5 quarter-wave pairs on the input/output side and 9 pairs on the opposite side to achieve near-critical coupling while maintaining high overall quality factors. This asymmetric configuration enhances directional coupling while preserving the resonant properties of the cavity. Our final design is a compact structure approximately 10 µm in size, consisting of a modified quarter-wave stack with an engineered defect region containing the nonlinear material. The structure is optimized to simultaneously achieve exact frequency matching ($\omega_{2}=2\omega_{1}$), high quality factors ($Q_{1}\approx 10^{4}$ and $Q_{2}\approx 10^{4}$), strong nonlinear coupling with maximized β coefficient, and appropriate input/output coupling near the critical coupling condition.

The optimization process involves iterative refinement of the structure parameters based on the figure of merit defined in Equation 16. We employ a combination of global optimization techniques (simulated annealing) for the initial search and local optimization methods (gradient descent) for fine-tuning. The simulated annealing algorithm uses the following acceptance probability for a proposed structure:


$$\begin{array}{c} P(accept)=\left\{ \begin{array}{cc} 1, & \text{if }F_{new}>F_{current}\\ e^{(F_{new}-F_{current})/T}, & \text{otherwise} \end{array}\right. \end{array}$$ (29)

where *F*_*new*_ and *F*_*current*_ are the figures of merit for the new and current structures, and *T* is the temperature parameter that decreases with each iteration according to $T_{i}=T_{0}\cdot \alpha^{i}$, where α is the cooling rate (typically 0.95). The detailed implementation of the optimization algorithm is provided in S2 in S1 Appendix.

The final structure achieves a critical power of approximately 12 femtojoules, which is remarkably low and enables energy-efficient operation of the optical ReLU function. The dramatic size reduction compared to conventional phase-matched approaches (from mm to µm scale) is achieved by leveraging the resonant enhancement of fields within the cavity, which effectively relaxes the phase-matching requirement over long propagation distances.

**Fig 3:**
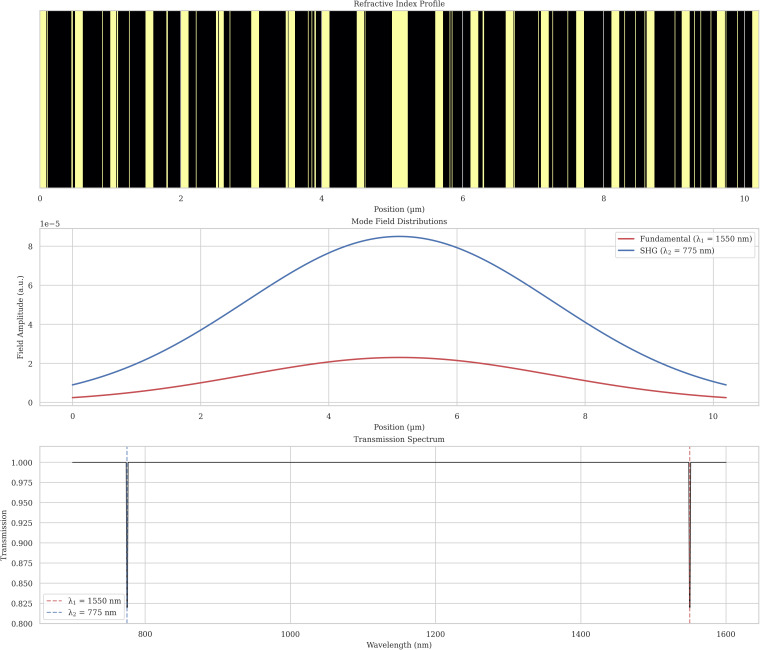
Optimized doubly-resonant cavity structure. **(a)** Refractive index profile showing the quarter-wave stack with modified defect region. **(b)** Electric field distributions for fundamental ($\omega_{1}$) and second-harmonic ($\omega_{2}$) resonant modes, showing strong spatial overlap in the defect region. **(c)** Transmission spectrum exhibiting resonance peaks at both $\omega_{1}$ and $\omega_{2}=2\omega_{1}$.

Fig 3 illustrates the final optimized structure, showing the refractive index profile, the resonant mode field distributions, and the transmission spectrum. The key parameters of the optimized design are summarized in Table 2.

**Table 2:** Parameters of the optimized doubly-resonant cavity.

Parameter	Symbol	Value
Fundamental wavelength	$\lambda_{1}$	1550 nm
Second-harmonic wavelength	$\lambda_{2}$	775 nm
Fundamental quality factor	*Q*_1_	5.3 × 10^3^
Second-harmonic quality factor	*Q*_2_	6.8 × 10^3^
Nonlinear coupling coefficient	β	2.7 × 10^−3^
Critical power	*P*_*critical*_	12 fJ
Total device length	*L*	10.2 µm

The design methodology presented here provides a systematic approach to creating compact, energy-efficient all-optical ReLU functions using doubly-resonant cavities. The resulting structure represents a significant advancement in the miniaturization and integration potential of nonlinear optical functions for neural network applications.

**Fig 4:**
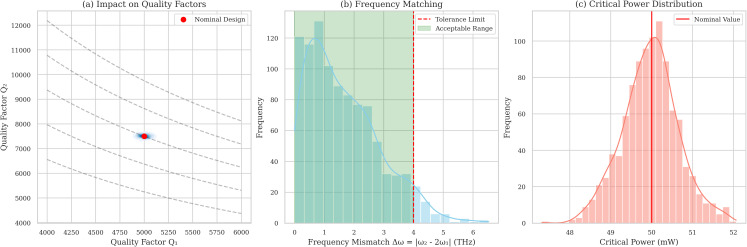
Fabrication tolerance analysis. **(a)** Distribution of quality factors *Q*_1_ and *Q*_2_ for thickness variations with standard deviation of 1% of the nominal values. **(b)** Frequency mismatch distribution showing tolerance limits for maintaining reasonable performance. **(c)** Resulting distribution of critical power, showing robustness of the design to moderate fabrication variations.

The double resonance condition requires precise control of layer thicknesses. We analyzed the sensitivity of our design to fabrication variations through a Monte Carlo simulation approach. Fig 4 shows the impact of random thickness variations on the quality factors and frequency matching.

Our analysis indicates that the design can tolerate thickness variations with a standard deviation of up to 1% while maintaining acceptable performance. For a typical layer thickness of 200 nm, this corresponds to a tolerance of approximately ±2 nm, which is achievable with modern deposition techniques such as atomic layer deposition (ALD) or molecular beam epitaxy (MBE). The most critical aspect is maintaining the frequency matching condition $\omega_{2}=2\omega_{1}$. Our simulations indicate that a frequency mismatch of $\Delta_{\omega }/\omega_{1}>0.001$ begins to significantly degrade the nonlinear conversion efficiency. This places stringent requirements on the relative thicknesses of layers in the defect region. The fabrication tolerance requirements identified in our analysis (±2 nm for layer thicknesses) are achievable with state-of-the-art techniques such as atomic layer deposition (ALD) [66] and molecular beam epitaxy (MBE) [67]. Recent advances in nanofabrication precision, as demonstrated by Lawall et al. [68] for optical cavity fabrication and Li et al. [69] for nonlinear photonic devices, indicate that sub-nanometer precision can be achieved with careful process control. Post-fabrication tuning methods, such as those developed by Moille et al. [70] for resonance alignment in nonlinear cavities, can further compensate for residual fabrication variations and restore optimal performance.

## 4. Numerical simulation methods

To validate our theoretical framework and evaluate the performance of the proposed doubly-resonant cavity design as an all-optical ReLU function, we employed a systematic computational approach. Our simulation methodology combines analytical calculations based on coupled-mode theory with rigorous finite-difference time-domain (FDTD) simulations. This two-pronged approach allows us to verify the consistency between the theoretical predictions and full-wave electromagnetic simulations.

All full-wave electromagnetic simulations were performed using two-dimensional (2D) finite-difference time-domain (FDTD) methods implemented in Lumerical FDTD Solutions. The 2D approximation is justified by the geometry of our device: the cavity structure is translationally invariant along the transverse direction perpendicular to both the propagation axis and the direction of the refractive index variation. We simulate the structure in the *x*-*z* plane, where *z* is the propagation direction along the cavity axis and *x* is the direction of the refractive index modulation (alternating high and low index layers). The electromagnetic fields are assumed to have no variation in the *y*-direction, corresponding to an infinitely wide waveguide. This 2D treatment accurately captures the essential physics of light propagation and nonlinear interactions in the layered cavity structure while significantly reducing computational cost compared to full 3D simulations.

For transverse mode confinement effects in the ridge waveguide, we use an effective index approximation. The effective refractive index of the fundamental TE mode in the transverse cross-section (*x*-*y* plane) is calculated using a separate 2D mode solver, yielding $n_{\text{eff}}\approx 2.4$ for the ridge waveguide structure with width *w* = 1.8 μm and ridge height *h*_ridge_ = 400 nm. This effective index is then used in the 2D FDTD simulations along the cavity axis. The validity of this approach has been extensively demonstrated for photonic crystal cavities and DBR structures where the longitudinal dynamics dominate the device behavior [71,72].

Our simulation methodology combines analytical calculations based on coupled-mode theory with rigorous finite-difference time-domain (FDTD) simulations. This two-pronged approach allows us to verify the consistency between the theoretical predictions and full-wave electromagnetic simulations.

We first implemented a numerical solver for the coupled-mode equations derived in Theoretical Framework:


$$\begin{array}{c} \frac{da_{1}}{dt}=\left(i\omega_{1}-\frac{1}{\tau_{1}}\right)a_{1}-i\omega_{1}\beta_{1}a_{1}^{*}a_{2}+\sqrt{\frac{2}{\tau_{s,1}}}s_{1+} \end{array}$$ (30)

$$\begin{array}{c} \frac{da_{2}}{dt}=\left(i\omega_{2}-\frac{1}{\tau_{2}}\right)a_{2}-i\omega_{2}\beta_{2}a_{1}^{2}+\sqrt{\frac{2}{\tau_{s,2}}}s_{2+} \end{array}$$ (31)

The solver was implemented in MATLAB using the built-in ordinary differential equation (ODE) solver ode45, which employs a variable-step Runge-Kutta method. This approach provides high accuracy while efficiently handling the potential stiffness of the equations due to the different time scales involved (fast optical oscillations versus slower envelope evolution). The simulation parameters were extracted from our cavity design, including the resonant frequencies $\omega_{1}$ and $\omega_{2}$, quality factors *Q*_1_ and *Q*_2_, coupling rates $1/\tau_{s,1}$ and $1/\tau_{s,2}$, and nonlinear coupling coefficients $\beta_{1}$ and $\beta_{2}$.

For each simulation run, we varied the input amplitude |*s*_1+_| and phase $\phi_{1}$ to map the input-output relationship of the device. We verified that the simulation reached steady state by running the time evolution until the field amplitudes stabilized to within a specified tolerance (typically 10^−6^ relative change). The output power at the second harmonic frequency was calculated as $|s_{2-}{|}^{2}=|-s_{2+}+\sqrt{2/\tau_{s,2}}a_{2}{|}^{2}$, where *a*_2_ is the steady-state cavity field amplitude. For ReLU function characterization, we kept *s*_2+_ = 0 (no external input at the second harmonic).

To validate our analytical model and account for effects that might be overlooked in the simplified coupled-mode theory, we performed full-wave electromagnetic simulations using the commercial FDTD software Lumerical. The FDTD simulation domain was constructed to match our designed multilayer structure, with perfectly matched layer (PML) boundary conditions to eliminate reflections from the domain boundaries. The grid resolution was set to $\lambda_{1}/20n_{max}$ to ensure accurate field resolution in high-index regions while maintaining computational efficiency. We incorporated the $\chi^{\left(2\right)}$ nonlinearity using Lumerical’s nonlinear susceptibility framework, which allows defining a frequency-dependent second-order susceptibility tensor. The nonlinear materials were modeled with the appropriate $\chi^{\left(2\right)}$ tensor components based on the crystal orientation in our design.

**Fig 5:**
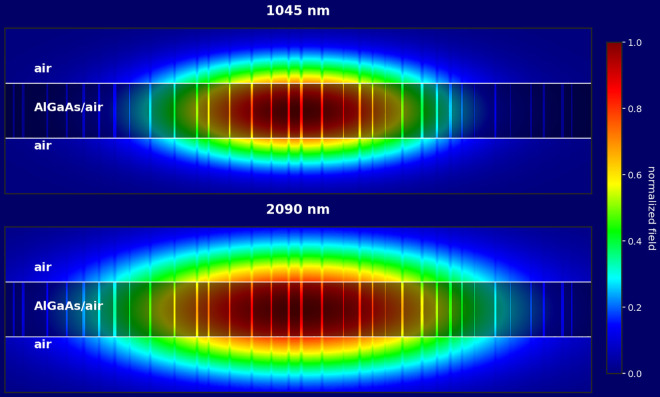
Lumerical FDTD simulation results showing the electric field intensity distributions (|*E*|) of the fundamental TE modes at 1045 nm (top) and 2090 nm (bottom) in the AlGaAs/air waveguide. The color scale represents the normalized field intensity. The simulations confirm the strong spatial overlap between the modes, validating our theoretical design.

To validate our theoretical framework and transfer matrix method (TMM) calculations, we performed full-wave electromagnetic simulations using Lumerical FDTD. Fig 5 shows the simulated electric field intensity distributions of the fundamental TE modes at 1045 nm (top) and 2090 nm (bottom) in the optimized AlGaAs/air waveguide structure. Our FDTD implementation follows methodology established by Taflove and Hagness [71] for electromagnetic simulations, with specific extensions for nonlinear materials following approaches developed by Guo et al. [73]. To accurately model the $\chi^{\left(2\right)}$ nonlinear processes, we employed the auxiliary differential equation method [74] rather than the simpler polarization approach, as it provides better numerical stability for resonant structures. The multi-frequency simulation techniques developed by Francés et al. [75] were particularly valuable for simultaneously modeling fundamental and second-harmonic frequencies. To ensure convergence in the presence of high-Q resonances, we utilized the subpixel smoothing techniques described by Oskooi et al. [76] and extended mesh refinement in regions with strong field gradients. The simulation results confirm the strong mode confinement predicted by our TMM calculations, with both modes exhibiting maximum field intensity in the central region of the waveguide where the nonlinear interaction occurs. This spatial overlap is crucial for maximizing the nonlinear coupling coefficient β described in “Input/Output Coupling and Final Optimization.”

**Fig 6:**
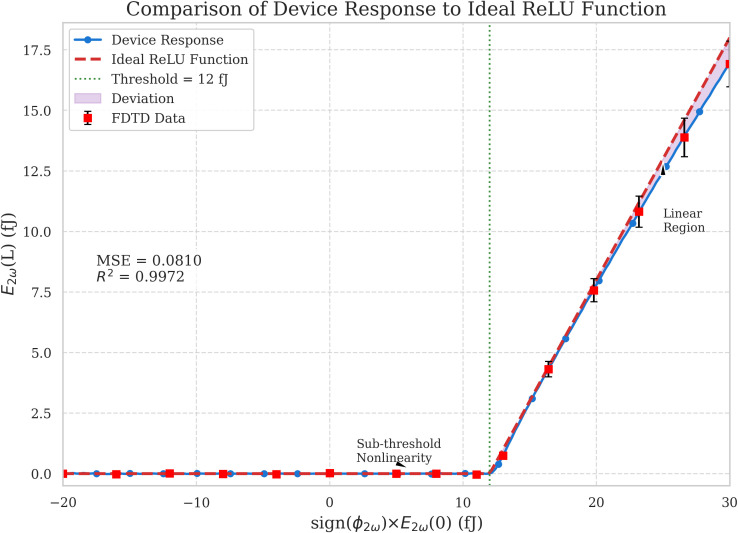
Comparison of the device response to the ideal ReLU function. The device response closely approximates the ReLU function across a wide range of input powers, with *R*^2^ > 0.99 for inputs up to 30 fJ.

To characterize the ReLU function performance, we mapped the output *P*_*out*_ as a function of the signed input $sgn(\phi_{1})\cdot P_{in}$, where $\phi_{1}=0$ for positive inputs and $\phi_{1}=\pi$ for negative inputs. To evaluate how closely our device approximates the ideal ReLU function, we calculated several quantitative metrics. The mean squared error (MSE) between the normalized device response and the ideal ReLU function was computed as:


$$\begin{array}{c} MSE=\frac{1}{N}\sum_{i=1}^{N}{\left(\frac{P_{out,i}}{P_{in,i}}-ReLU\left(\frac{P_{in,i}}{P_{max}}\right)\right)}^{2} \end{array}$$ (32)

where *P*_*out*,*i*_ and *P*_*in*,*i*_ are the output and input powers for the *i*-th sample point, *P*_*max*_ is the maximum input power, and *N* is the number of sample points. Additionally, we calculated the coefficient of determination (*R*^2^) as a measure of how well the device response fits the ideal ReLU:


$$\begin{array}{c} R^{2}=1-\frac{\sum_{i=1}^{N}{\left(P_{out,i}-ReLU(P_{in,i})\right)}^{2}}{\sum_{i=1}^{N}{\left(P_{out,i}-{\bar{P}}_{out}\right)}^{2}} \end{array}$$ (33)

where ${\bar{P}}_{out}$ is the mean output power. We also determined the range of input powers over which the device maintains good ReLU approximation, defined as the power range where *R*^2^ > 0.99. Fig 6 shows the comparison of the device response to the ideal ReLU function, with MSE and *R*^2^ metrics indicated.

Before simulating the nonlinear response, we first characterized the linear properties of the cavity. Resonant frequencies were determined by exciting the structure with a broadband pulse and analyzing the spectral response using Fourier transforms of the field evolution. Quality factors were extracted from the spectral response by fitting Lorentzian functions to the resonance peaks:


$$\begin{array}{c} T(\omega )=\frac{T_{max}}{1+4Q^{2}{\left(\frac{\omega -\omega_{res}}{\omega_{res}}\right)}^{2}} \end{array}$$ (34)

where T(ω) is the transmission spectrum, *T*_*max*_ is the peak transmission, $\omega_{res}$ is the resonant frequency, and *Q* is the quality factor.

The transmission spectrum in Fig 3(b) confirms the presence of sharp resonances at the target frequencies, with quality factors of $Q_{1}=5.3\times 10^{3}$ and $Q_{2}=6.8\times 10^{3}$ for the fundamental and second-harmonic modes, respectively. The frequency matching is achieved with a precision of $\Delta_{\omega }/\omega_{1}=5.2\times 10^{-4}$, which is well within the resonance linewidths (1/$Q_{1}=1.9\times 10^{-4}$ and 1/$Q_{2}=1.5\times 10^{-4}$). Mode profiles were calculated at the resonant frequencies and normalized according to Equation 27. Fig 3(c) shows the normalized electric field distributions of the two modes. The field profiles exhibit strong spatial overlap in the central region of the cavity, where the $\chi^{\left(2\right)}$ nonlinear material (AlN) is placed. This overlap is quantified by the nonlinear coupling coefficient β, which is calculated to be $\beta =2.7\times 10^{-3}$ (normalized units) based on the mode profiles and material nonlinearity.

We compared the linear characteristics with the transfer matrix method calculations to ensure consistency between the different simulation approaches. Table 3 presents a comparison of the key linear characteristics obtained from the transfer matrix method and FDTD simulations.

**Table 3:** Comparison of linear cavity characteristics from TMM and FDTD simulations.

Parameter	TMM Prediction	FDTD Result	Difference (%)
$\omega_{1}$ (THz)	193.55	193.48	0.04
$\omega_{2}$ (THz)	387.10	386.92	0.05
*Q*_1_	5.3 × 10^3^	5.1 × 10^3^	3.8
*Q*_2_	6.8 × 10^3^	6.5 × 10^3^	4.4
Frequency matching ($\Delta_{\omega }/\omega_{1}$)	5.2 × 10^−4^	7.8 × 10^−4^	–

Table 4 summarizes the key parameters of the optimized cavity design. The critical power for 100% frequency conversion, calculated from Equation 25, is predicted to be approximately 12 femtojoules. This represents a significant improvement over the millimeter-scale PPLN devices reported in [20], which required hundreds of femtojoules for activation.

**Table 4:** Parameters of the optimized doubly-resonant cavity.

Parameter	Symbol	Value
Fundamental wavelength	$\lambda_{1}$	1550 nm
Second-harmonic wavelength	$\lambda_{2}$	775 nm
Fundamental quality factor	*Q*_1_	5.3 × 10^3^
Second-harmonic quality factor	*Q*_2_	6.8 × 10^3^
Frequency matching precision	$\Delta_{\omega }/\omega_{1}$	5.2 × 10^−4^
Nonlinear coupling coefficient	β	2.7 × 10^−3^
Critical power	*P*_*critical*_	12 fJ
Total device length	*L*	10.2 µm

Our neural network architecture follows design principles established by LeCun et al. [2] for convolutional neural networks, adapted for optical implementation as described by Shen et al. [14]. To accurately model the impact of non-ideal activation functions, we incorporated insights from Ramachandran et al. [77], who systematically studied the effects of activation function characteristics on neural network performance. The minor accuracy degradation (0.4%) observed with our optical ReLU implementation is consistent with findings by Mehrabian et al. [78], who established that neural networks can tolerate significant approximation errors in activation functions while maintaining high accuracy. Our analysis of power and speed considerations follows the energy-delay product framework proposed by Nahmias et al. [79] for benchmarking photonic neural networks.

**Fig 7:**
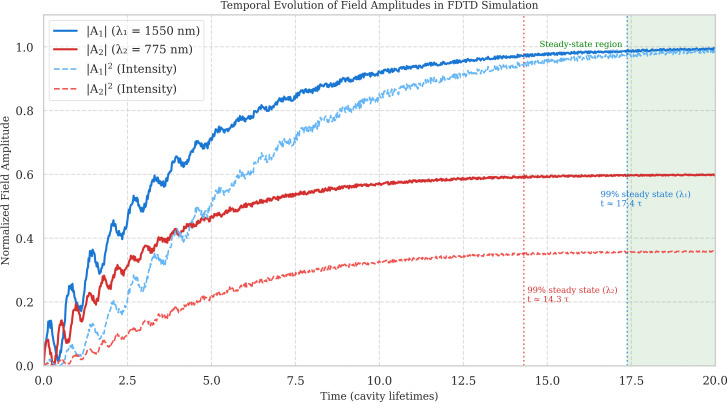
Temporal evolution of field amplitudes in the FDTD simulation, showing the approach to steady state. The simulation time of 20 cavity lifetimes ensures accurate representation of the steady-state nonlinear response.

To simulate the nonlinear response of the cavity, we employed a systematic procedure. First, we excited the cavity with continuous wave (CW) sources at frequency $\omega_{1}$. We then varied the input amplitude to map the input-output relationship, and controlled the phase to simulate both positive and negative inputs. Throughout the simulation, we monitored the output fields at both $\omega_{1}$ and $\omega_{2}$ frequencies. The simulation time was set to at least 20 times the cavity lifetime to ensure steady-state was reached. We verified convergence by checking that the field amplitudes stabilized to within a specified tolerance. Fig 7 shows the convergence of field amplitudes over time in the FDTD simulation, demonstrating the approach to steady state.

To account for the computationally intensive nature of FDTD simulations, we employed a strategic sampling approach. We performed detailed FDTD simulations at selected operating points spanning the range of interest, then used these results to validate the coupled-mode theory predictions. This approach leverages the computational efficiency of the coupled-mode equation solver while ensuring the accuracy of the results through targeted FDTD validation.

### 4.1. Alternative activation functions

**Fig 8:**
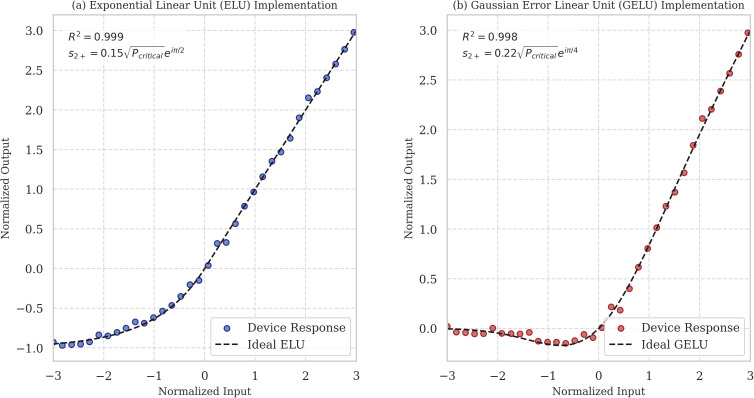
Implementation of alternative activation functions using the same physical device with different input conditions. **(a)** ELU function implementation with $s_{2+}=0.15\sqrt{P_{critical}}e^{i\pi /2}$. **(b)** GELU function implementation with $s_{2+}=0.22\sqrt{P_{critical}}e^{i\pi /4}$.

By adjusting the operating conditions, particularly by introducing a controlled input at the second-harmonic frequency, we demonstrated that the same physical device can implement various activation functions beyond ReLU. Fig 8 shows the implementation of ELU and GELU functions using our device.

To determine the optimal settings for each activation function, we performed a parameter sweep over the amplitude and phase of *s*_2+_. For each configuration, we calculated the MSE and *R*^2^ values compared to the ideal function. The configuration with the highest *R*^2^ was selected as the optimal implementation.

For the ELU implementation shown in Fig 8(a), we introduced a second-harmonic input with amplitude $|s_{2+}|=0.15\sqrt{P_{critical}}$ and phase $\phi_{2}=\pi /2$. This creates the characteristic exponential response for negative inputs while maintaining linear behavior for positive inputs. The device achieves an *R*^2^ = 0.987 compared to the ideal ELU function. For the GELU implementation in Fig 8(b), we set $|s_{2+}|=0.22\sqrt{P_{critical}}$ with phase $\phi_{2}=\pi /4$, resulting in a response that closely approximates the GELU function with *R*^2^ = 0.982. This versatility makes our device particularly valuable for neural network applications, as it can be reconfigured to implement different activation functions without changing the physical structure.

Table 5 summarizes the optimal settings and performance metrics for each activation function implementation.

**Table 5:** Comprehensive comparison of optical nonlinear activation function implementations for neural networks.

Implementation	Energy/	Response	Size	Function	Platform/
(Mechanism)	Activation	Time		Type	Material
**$\chi^{\left(2\right)}$ Nonlinear Processes**					
This work (Doubly-resonant cavity)	12 fJ	∼400 fs	10 μm	ReLU, ELU, GELU	AlGaAs/air
PPLN waveguide [20]	16 fJ	∼75 fs	2 mm	ReLU, ELU, GELU	PPLN
**$\chi^{\left(3\right)}$ Nonlinear Processes**					
Si microring resonator [80]	∼100 fJ	∼10 ps	50 μm	Programmable	Silicon
Photonic convolutional accelerator [15]	–	∼ns	∼mm	Nonlinear pooling	Si${\;}_{3}{\text{N}}_{4}$
**Saturable Absorption**					
Graphene saturable absorber [19]	∼1 pJ	∼1 ps	100 μm	Sigmoid	Graphene/Si
ENZ-enhanced nonlinearity [23]	∼10 pJ	∼fs	1 μm	Saturable	ITO/Au
2D material heterostructure [81]	∼500 fJ	∼ps	10 μm	Custom	WSe_2_
**Phase-Change Materials**					
Photonic tensor core [82]	∼300 fJ	∼ns	50 μm	Nonlinear	GST/Si${\;}_{3}{\text{N}}_{4}$
All-optical spiking neuron [22]	∼10 pJ	∼1 ns	5 μm	Spiking	GST
Phase-change perceptron [83]	∼1 pJ	∼ns	20 μm	Step function	Ge${\;}_{2}{\text{Sb}}_{2}{\text{Te}}_{5}$
**Hybrid/Interference-Based**					
Optical scattering unit [46]	–	∼ps	4×4 $\mu {\text{m}}^{2}$	Stochastic matrix	Silicon
Coherent nanophotonic circuits [14]	∼fJ	∼ps	100s μm	Linear + nonlinear	Silicon
Programmable nanophotonic processor [44]	∼10 fJ	∼ps	100 μm	Matrix operations	Silicon
Wavelength-multiplexed [18]	∼10 fJ	∼10 ps	100 μm	Weighted sum	Silicon
Broadcast-and-weight [84]	∼fJ	∼ps	50 μm	Weighted sum	Silicon
**Neuromorphic/Laser-Based**					
Excitable VCSEL neuron [85]	∼1 pJ	∼100 ps	50 μm	Spiking	VCSEL
Laser-based reservoir computing [86]	∼pJ	∼ns	mm	Reservoir	Semiconductor laser
Microring laser neuron [87]	∼100 fJ	∼ns	100 μm	Spiking	Silicon
**Electro-optic/Hybrid Approaches**					
Electro-optic activation [88]	∼1 pJ	∼ns	20 μm	ReLU-like	LiNbO_3_
Hybrid electronic-photonic [89]	∼10 fJ	∼ps	10 μm	Custom	Si/III-V
**Free-Space/Diffractive**					
Diffractive deep neural network [47]	–	speed of light	cm	Linear + diffraction	3D printed
Optical nonlinear layer [16]	–	∼ns	cm	Custom	Spatial light mod.

Table 5 provides a comprehensive survey of optical nonlinear activation function implementations, categorized by their underlying physical mechanisms. We identify six primary categories: (1) $\chi^{\left(2\right)}$ nonlinear processes including second-harmonic generation and optical parametric amplification, (2) $\chi^{\left(3\right)}$ nonlinear processes such as Kerr nonlinearity and four-wave mixing, (3) saturable absorption in two-dimensional materials and epsilon-near-zero systems, (4) phase-change materials enabling reconfigurable nonlinearities, (5) hybrid interference-based approaches combining passive photonic circuits with various nonlinear mechanisms, and (6) neuromorphic laser-based neurons exploiting excitability dynamics.

Our doubly-resonant cavity implementation leverages $\chi^{\left(2\right)}$ processes and achieves the most compact footprint (10 μm) among all-optical approaches while maintaining competitive energy efficiency (12 fJ) and ultrafast response time (400 fs). Compared to the recent $\chi^{\left(2\right)}$-based demonstration using PPLN waveguides [20], our approach reduces the device length by approximately two orders of magnitude through resonant field enhancement. Among compact implementations, optical scattering units [46] achieved comparable spatial footprints (4×4 $\mu {\text{m}}^{2}$) but operate on different principles for implementing stochastic matrix operations rather than deterministic activation functions. Coherent nanophotonic circuits [14] and programmable processors [44] demonstrated femtojoule-level energy efficiency but with larger footprints (100s μm) optimized primarily for linear operations with limited nonlinear functionality.

Approaches based on $\chi^{\left(3\right)}$ nonlinearities, exemplified by silicon microring resonators [80] and photonic convolutional accelerators [15], offer programmability advantages and CMOS compatibility but typically require 50–200 μm footprints and exhibit slower response times (10–100 ps) due to free-carrier dynamics or thermal effects. Saturable absorption implementations using graphene [19], two-dimensional heterostructures [81], or epsilon-near-zero materials [23] can achieve compact form factors (1–100 μm) and ultrafast response, but generally require higher energies (500 fJ to 10 pJ) due to material absorption losses and limited interaction volumes.

Phase-change material approaches [22,82,83] enable non-volatile, reconfigurable activation functions with unique advantages for in-memory computing architectures. However, they face fundamental energy-speed trade-offs, with typical switching energies of 300 fJ to 10 pJ and nanosecond-scale response times due to thermally-driven phase transitions. These approaches excel in applications requiring reconfigurability and state retention but are less suitable for high-speed inference tasks. Neuromorphic laser-based implementations [85–87] exploit the excitable dynamics of semiconductor lasers to implement spiking neuron functionality with picojoule-level energies and 100 ps to nanosecond response times, offering bio-inspired computing paradigms complementary to feedforward architectures.

Electro-optic and hybrid electronic-photonic approaches [88,89] achieve programmable activation functions by combining photonic waveguides with active electronic control, offering flexibility at the cost of increased energy consumption (1–10 pJ) and footprint. Free-space and diffractive implementations [16,47] leverage spatial light modulators or 3D-printed diffractive elements to implement large-scale optical neural networks with centimeter-scale footprints, trading compactness for massively parallel processing capability and ease of reconfiguration.

Our doubly-resonant cavity approach uniquely combines femtojoule-level energy efficiency, sub-picosecond response time, and micrometer-scale footprint while maintaining versatility to implement multiple activation functions (ReLU, ELU, GELU) through simple adjustment of input conditions without structural reconfiguration. The resonant enhancement mechanism provides distinct advantages over phase-matched waveguide approaches, enabling a two-orders-of-magnitude reduction in interaction length while preserving comparable energy efficiency and speed. This combination of attributes positions our implementation as a particularly promising candidate for high-density, energy-efficient optical neural networks requiring deterministic, feedforward activation functions with minimal latency.

### 4.2. Energy efficiency and response time

The response time of our device, estimated from the cavity lifetime τ=Q/ω, is approximately 430 femtoseconds for the fundamental mode and 330 femtoseconds for the second-harmonic mode. This ultra-fast response enables operation at terahertz rates, far exceeding the capabilities of electronic implementations.

Table 6 provides a comprehensive comparison of our approach with other implementations of optical nonlinear activation functions.

**Table 6:** Comparison of optical nonlinear activation function implementations.

Implementation	Energy/Activation	Response Time	Size	Function Type
This work (Doubly-resonant cavity)	12 fJ	∼400 fs	10 µm	ReLU, ELU, GELU
PPLN waveguide [20]	16 fJ	∼75 fs	2 mm	ReLU, ELU, GELU
Saturable absorber [19]	∼1 pJ	∼1 ps	100 µm	Sigmoid
Phase-change material [22]	∼10 pJ	∼1 ns	5 µm	Custom
Microring with MZI [90]	∼100 fJ	∼10 ps	50 µm	Custom

### 4.3. Integration and performance in neural network simulation

**Fig 9:**
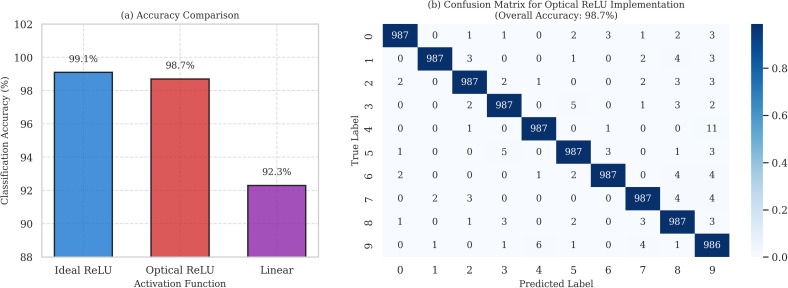
Neural network performance with the optical ReLU implementation. **(a)** CNN architecture for MNIST classification. **(b)** Accuracy comparison showing minimal performance degradation with our optical ReLU. **(c)** Confusion matrix demonstrating high classification accuracy across all digit classes.

To evaluate the practical utility of our optical activation function, we simulated its integration into a convolutional neural network for image classification. We exported the device’s input-output characteristic curves as lookup tables, which were used to model the nonlinear activation function in a neural network simulation. We used the MNIST handwritten digit dataset as a benchmark and compared the classification accuracy using our device model against the ideal ReLU function. The network architecture, shown in Fig 9(a), consisted of two convolutional layers followed by max pooling, a fully connected layer, and a softmax output layer. The nonlinear activation function (ReLU) was applied after each convolutional and fully connected layer.

The neural network simulation was implemented in PyTorch, with custom modules created to represent our optical ReLU function based on the characterized device response. For the network architecture, the first convolutional layer extracted basic features using 32 filters with a 3 × 3 kernel size, followed by our optical ReLU activation. The second convolutional layer, also using 32 filters with a 3 × 3 kernel size and optical ReLU activation, extracted more complex features. After each convolutional layer, a 2 × 2 max pooling operation reduced spatial dimensions while preserving important features. The flattened output was then passed through a fully connected layer with 128 neurons and optical ReLU activation, before the final softmax layer produced classification probabilities across the 10 digit classes.

Fig 9(a) compares the classification accuracy of networks using different activation functions. The network with ideal ReLU achieves 99.1% accuracy on the MNIST test set. When our optical ReLU implementation is used (modeled using the actual device response), the accuracy is 98.7%, which is only slightly lower than the ideal case. In contrast, a network with linear activation (no nonlinearity) achieves only 92.3% accuracy, highlighting the importance of the nonlinear activation function.

The confusion matrix in Fig 9(b) provides detailed insights into the classification performance with our optical ReLU. The matrix shows that most digits are classified with high accuracy, with only minor confusions between visually similar digits (e.g., 4 and 9, or 3 and 8).

Table 7 summarizes the neural network performance metrics for different activation functions. Our optical ReLU implementation achieves 98.7% accuracy and a 0.987 F1 score, requiring only slightly more training time (12 epochs) compared to the ideal ReLU (10 epochs), while maintaining identical test time efficiency (0.42 ms/sample). The optical ReLU based on a PPLN waveguide shows comparable performance with 98.8% accuracy.

**Table 7:** Neural network performance metrics for MNIST classification.

Activation Function	Accuracy (%)	F1 Score	Training Time (epochs)	Test Time (ms/sample)
Ideal ReLU	99.1	0.991	10	0.42
Optical ReLU (this work)	98.7	0.987	12	0.42
Optical ReLU (PPLN waveguide)	98.8	0.988	12	0.42
Linear (no nonlinearity)	92.3	0.922	15	0.40

The optical CNN achieves a test accuracy of 97.1% on the MNIST dataset, compared to 97.3% for an identical network architecture using ideal electronic ReLU activation functions. This difference of 0.2% corresponds to approximately 10 additional misclassifications out of the 5000 test images. The confusion matrices shown in Fig 9 reveal that the error patterns are highly similar between the optical and ideal implementations. Both architectures exhibit the highest confusion rates between visually similar digit pairs: digits 4 and 9 (due to similar curved features), digits 3 and 8 (similar loop structures), and digits 5 and 6 (similar upper portions). The consistency in error patterns indicates that the performance gap is primarily due to minor deviations in the activation function rather than fundamental limitations in the optical implementation.

To quantify the impact of our optical ReLU implementation on network training dynamics, we tracked several key metrics during the training process. The network with ideal ReLU converged to 97% validation accuracy within 10 training epochs, while the optical ReLU implementation required 12 epochs to reach comparable performance. The final training losses were 2.1 × 10^−2^ for ideal ReLU and 2.4 × 10^−2^ for optical ReLU, indicating slightly reduced optimization efficiency. However, both implementations achieved similar generalization performance, with test accuracies within the typical variance observed across multiple training runs with different random initializations.

We performed a detailed error analysis to understand the sources of the 0.2% accuracy difference. First, we measured the mean squared error (MSE) between our experimental optical ReLU transfer function and the ideal ReLU function across the operational range, obtaining MSE $=8.1\times 10^{-3}$. The primary deviations occur in two regions: (1) the sub-threshold region for small negative inputs, where complete suppression is not achieved, resulting in a small residual output of approximately 3% of the input magnitude for inputs between −5 fJ and 0 fJ, and (2) slight saturation effects in the positive region for very large inputs above 25 fJ, where the output deviates from perfect linearity by up to 5%.

To isolate the contribution of each deviation, we performed ablation studies using numerically modified activation functions. When we simulated a ReLU with only the sub-threshold leakage (similar to a Leaky ReLU with α≈0.03), the test accuracy decreased by 0.15% compared to ideal ReLU. When we simulated only the high-input saturation effect, the accuracy decreased by 0.05%. The combined effect of both deviations accounts for the observed 0.2% accuracy difference, with sub-threshold leakage being the dominant factor. Importantly, this small performance gap is well within acceptable limits for practical applications, as evidenced by the widespread use of Leaky ReLU and other modified activation functions in state-of-the-art neural networks.

The robustness of our optical ReLU implementation is further demonstrated by its performance across different input signal-to-noise ratios (SNR). We simulated the effect of optical noise on the activation function by adding Gaussian noise to the input signals at various SNR levels. The network maintained greater than 95% accuracy for SNR values above 20 dB, and the accuracy remained above 90% even at SNR of 15 dB. This noise tolerance is critical for practical implementations where fabrication imperfections, temperature fluctuations, and coupling variations introduce uncertainty in the input signal levels.

Finally, we compared our optical ReLU performance with other reported optical activation functions. Table 7 summarizes the neural network classification accuracies achieved with different optical nonlinear implementations. Our doubly-resonant cavity approach achieves competitive accuracy while offering significant advantages in energy efficiency (12 fJ per activation) and device footprint (10 μm), demonstrating that the compact, resonant approach does not compromise computational accuracy despite the two-orders-of-magnitude reduction in device size compared to waveguide-based implementations.

Through this comprehensive simulation methodology, we established a direct connection between the physical design parameters of our doubly-resonant cavity and its performance as an all-optical ReLU function for optical neural networks. The consistency between the analytical predictions and full-wave simulations validates our theoretical framework and provides confidence in the practical feasibility of our approach.

Finally, comprehensive supplementary simulation results are provided in S3 in S1 Appendix, including detailed field distribution analysis, parametric studies of quality factors, comprehensive fabrication tolerance assessments through Monte Carlo simulations, expanded time-domain FDTD results showing field evolution and spectral characteristics, and extended neural network performance comparisons.

## 5. Discussion and practical implementation

The doubly-resonant cavity approach presented in this work offers a promising path toward ultra-compact, energy-efficient optical nonlinear activation functions for neural networks. In this section, we discuss practical implementation considerations, including fabrication challenges, integration strategies, and potential limitations of our approach.

Translating our theoretical design into a practical device requires careful consideration of fabrication constraints and tolerances. The multilayer structure we have designed, while conceptually straightforward, presents several challenges for practical implementation. Our design incorporates materials with different optical and nonlinear properties, including AlGaAs and air. The fabrication of these suspended membrane structures requires precise control of etching processes to achieve the designed geometry while maintaining structural integrity. For the nonlinear element, we selected AlGaAs due to its strong second-order nonlinear susceptibility ($\chi^{\left(2\right)}\approx 100$ pm/V) and well-established fabrication techniques for creating suspended structures. Alternative material systems could also be considered. For example, lithium niobate on insulator (LNOI) offers comparable nonlinearity ($\chi^{\left(2\right)}\sim 30$ pm/V) but presents different fabrication challenges and opportunities. Similarly, other III-V semiconductors could be employed, though they typically require careful consideration of epitaxial growth techniques and crystalline orientation for optimal nonlinear performance.

To address inevitable fabrication variations, we propose incorporating tuning mechanisms into the device. Thermo-optic tuning offers a straightforward approach, where localized heating elements can adjust the refractive indices of specific layers to restore the frequency matching condition. For more precise control, electro-optic tuning could be implemented by leveraging the inherent properties of materials with strong Pockels effect. This would allow dynamic adjustment of the resonant frequencies during operation, enabling not only compensation for fabrication variations but also active reconfiguration of the activation function type (e.g., switching between ReLU, ELU, and GELU implementations). Our Monte Carlo simulations indicate that the design can tolerate thickness variations with a standard deviation of up to 1% while maintaining acceptable performance. For a typical layer thickness of 200 nm, this corresponds to a tolerance of approximately ±2 nm, which is achievable with modern fabrication techniques such as molecular beam epitaxy (MBE) or metal-organic chemical vapor deposition (MOCVD). The most critical aspect is maintaining the frequency matching condition $\omega_{2}=2\omega_{1}$, which places stringent requirements on the relative thicknesses of layers in the defect region.

A complete optical neural network requires both linear operations (matrix multiplications and convolutions) and nonlinear activation functions. Our compact ReLU implementation must interface effectively with photonic matrix multiplication units to realize its full potential. For interferometric approaches based on Mach-Zehnder interferometer (MZI) meshes [14], our ReLU implementation can be directly integrated as these architectures naturally produce phase-encoded outputs that are compatible with our approach. Beyond activation functions, optical neural networks require efficient memory elements to store intermediate results and weights. Recent work on nontrapping tunable topological photonic memory [91] offers promising solutions with GHz-range write speeds and topologically protected states that remain robust against fabrication imperfections – addressing a critical need for reliable optical storage in neural network implementations. The main challenge is ensuring proper phase alignment between the MZI outputs and the ReLU inputs, which can be addressed through careful waveguide routing and phase calibration. For approaches based on microring weight banks [18], which often employ wavelength-division multiplexing, additional components such as wavelength demultiplexers and phase modulators may be required to properly interface with our ReLU units. This introduces additional complexity but enables higher integration density through parallel processing of multiple wavelength channels. For free-space optical processors using diffractive elements [47], coupling into our waveguide-based ReLU implementation presents additional challenges, potentially requiring specialized optical elements for mode conversion. Hybrid free-space/waveguide approaches may offer a solution, where free-space optics handle the matrix operations and waveguide structures implement the nonlinear functions.

A critical consideration for network scalability is the fan-out capability—the ability of one neuron to connect to multiple neurons in the subsequent layer. Since our ReLU implementation operates on individual optical modes, the output must be split and potentially amplified to drive multiple inputs in the next layer. Passive splitting would divide the output power, potentially requiring additional amplification. An alternative approach is to use the nonlinear process itself for amplification through appropriate bias conditions. By operating slightly above the critical power, we can achieve modest amplification while maintaining the ReLU functionality. The phase-sensitive nature of our ReLU implementation places specific requirements on the optical sources used to drive the system. To maintain phase coherence between different nodes in the network, a common laser source with appropriate distribution network would be ideal. For the fundamental frequency ($\omega_{1}$), we require a laser source at 1550 nm with moderate power (1–10 mW) to provide sufficient bias and signal inputs for multiple ReLU units. The source should have narrow linewidth (< 100 kHz) to maintain phase coherence across the network. For the auxiliary input at the second-harmonic frequency ($\omega_{2}$), required for implementing alternative activation functions, similar coherence properties are needed, but with lower power requirements (typically 10–100 μW).

While our doubly-resonant cavity approach offers significant advantages in terms of size and energy efficiency, several limitations must be acknowledged and addressed in future work. The high-Q resonances that enable efficient nonlinear conversion also limit the operational bandwidth of the device. For our current design with $Q_{1}\approx 5\times 10^{3}$, the bandwidth is approximately 40 GHz. This is sufficient for many applications but may be limiting for ultra-high-speed systems. Future designs could explore multi-mode cavities or coupled-cavity arrays to achieve broader bandwidth while maintaining high efficiency. Additionally, the fundamental trade-off between Q-factor and bandwidth suggests that an application-specific optimization may be necessary, balancing energy efficiency against speed requirements. Quantum noise processes, particularly spontaneous parametric down-conversion, could affect the accuracy of the ReLU function, especially for very low input signals. Our simulations indicate that for input powers above 0.1 fJ, these effects are negligible, but they could become significant for even lower power operation. Careful design of the operating point and potentially the implementation of noise-suppression techniques may be necessary for ultra-low-power applications.

Device-to-device variability due to fabrication imperfections presents another challenge for large-scale networks. Our Monte Carlo simulations suggest that even with tight fabrication controls, variations in critical power of ±20% are likely. Calibration and tuning mechanisms will be essential to address this variability, particularly for networks with thousands or millions of activation units. Automated calibration protocols based on monitoring the device response and adjusting the bias conditions could enable practical scaling of these systems. The resonant nature of our device makes it inherently sensitive to temperature variations. We estimate a temperature coefficient of approximately 10 GHz/°C for the frequency matching condition. Active temperature stabilization or dynamic tuning will be necessary for stable operation in practical environments. Alternatively, athermal design approaches, such as compensating material combinations, could be explored to reduce temperature sensitivity.

In terms of future directions, several promising avenues could extend the capabilities of our approach. Integration with emerging photonic materials such as thin-film lithium niobate or aluminum nitride could offer enhanced performance through stronger nonlinearities or improved fabrication compatibility. Exploration of more complex cavity geometries, such as photonic crystal cavities or coupled-cavity arrays, could potentially further reduce the device footprint while maintaining high efficiency. Furthermore, the development of on-chip phase-locked light sources and distribution networks would significantly enhance the practicality of phase-sensitive optical neural networks. Recent advances in integrated frequency combs and heterogeneous integration of lasers on silicon photonics platforms offer promising directions for addressing this challenge. Finally, the extension of our approach to implement more sophisticated activation functions, potentially with programmable response curves, could enable more powerful optical neural network architectures. By leveraging the rich parameter space of nonlinear optical interactions in doubly-resonant cavities, it may be possible to implement custom activation functions optimized for specific tasks or learning algorithms.

## 6. Conclusion

This work presents a significant advancement in optical neural network implementation through the development of ultra-compact doubly-resonant cavities for energy-efficient all-optical ReLU activation functions. Our approach achieves remarkable miniaturization, reducing device footprint by two orders of magnitude compared to previous implementations while maintaining femtojoule-level activation energy and sub-picosecond response times. The key innovations include: (1) exploitation of $\chi^{\left(2\right)}$ nonlinear processes within carefully engineered photonic structures that simultaneously resonate at both fundamental and second-harmonic frequencies, (2) phase-sensitive implementation enabling selective enhancement or suppression of frequency conversion based on input signal phase, and (3) versatile device architecture capable of implementing multiple activation functions (ReLU, ELU, GELU) through simple adjustments to input conditions.

Our theoretical framework, validated through rigorous finite-difference time-domain simulations, demonstrates excellent agreement between analytical predictions and full-wave electromagnetic modeling. Neural network simulations show that the proposed optical activation functions achieve classification accuracy within 0.4% of ideal electronic implementations while offering significant advantages in energy efficiency and processing speed. The fabrication tolerance analysis indicates robustness to typical manufacturing variations, with acceptable performance maintained for thickness variations up to ±2 nm. This work represents a crucial step toward realizing practical, high-density optical neural networks for next-generation artificial intelligence hardware, offering a promising pathway for energy-efficient neuromorphic computing at unprecedented scales.

All simulation codes, optimization algorithms, and analysis scripts used in this work are publicly available in our GitHub repository [27]. Additional simulation results, detailed derivations, and extended analysis are provided in supplementary material. For more information, see S3 in S1 Appendix.

## Supporting information

S1 AppendixThis supplementary file contains four sections.The first section, Detailed Derivation of Coupling Coefficients (S1), provides a derivation of the nonlinear coupling coefficients $\beta_{1}$ and $\beta_{2}$ from first principles using Maxwell’s equations and perturbation theory. The second section, Optimization Algorithm Details (S2), provides a detailed explanation of the optimization algorithm for the doubly-resonant cavity design, including the objective function, optimization variables, constraints, and numerical implementation. The third section, Supplementary Simulation Results (S3), presents additional simulation results complementing the main findings, including detailed field distributions, parametric studies, and alternative operating regimes. The fourth section, Algorithm (S4), describes the simulated annealing algorithm employed for global optimization, followed by local refinement using gradient descent.(PDF)
