# Supplementary material for: All-optical doubly resonant cavities for energy-efficient ReLU function in nanophotonic deep learning
Source: PLoS One. 2026 Jun 17;21(6):e0345850. doi: 10.1371/journal.pone.0345850 (PMC13274909; doi:10.1371/journal.pone.0345850)
Supplement: S1 Appendix — The first section, Detailed Derivation of Coupling Coefficients (S1), provides a derivation of the nonlinear coupling coefficients β1 and β2 from first principles using Maxwell’s equations and perturbation theory. The second section, Optimization Algorithm Details (S2), provides a detailed explanation of the optimization algorithm for the doubly-resonant cavity design, including the objective function, optimization variables, constraints, and numerical implementation. The third section, Supplementary Simulation Results (S3), presents additional simulation results complementing the main findings, including detailed field distributions, parametric studies, and alternative operating regimes. The fourth section, Algorithm (S4), describes the simulated annealing algorithm employed for global optimization, followed by local refinement using gradient descent. (PDF) [file pone.0345850.s001.pdf]

Supplementary Material:
All-Optical Doubly Resonant Cavities for Energy-Efficient ReLU Function in Nanophotonic Deep Learning

Amirreza Ahmadnejad[1], Mohammad Mehrdad Asadi[1], Somayyeh Koohi[2*],

1 Department of Electrical Engineering Sharif University of Technology Tehran, Iran
2 Department of Computer Engineering , Sharif University of Technology Tehran, Iran

* koohi@sharif.edu

S1 Detailed Derivation of Coupling Coefficients

In Section 2.3, we introduced the nonlinear coupling coefficients β_1 and β_2 that quantify the interaction strength between the fundamental and second-harmonic modes via $\chi^{(2)}$ nonlinearity. Here, we provide a detailed derivation of these coefficients from first principles, starting with Maxwell's equations and using perturbation theory.

We begin with the wave equation for the electric field in a nonlinear medium:

$$\nabla \times \nabla \times \mathbf{E} - \frac{\omega^2}{c^2}\varepsilon(\mathbf{r})\mathbf{E} = \frac{\omega^2}{c^2}\mathbf{P}^{NL} \tag{S1}$$

where $\mathbf{E}$ is the electric field, ω is the frequency, $\varepsilon(\mathbf{r})$ is the linear dielectric function, c is the speed of light, and $\mathbf{P}^{NL}$ is the nonlinear polarization. For a $\chi^{(2)}$ nonlinear medium, the second-order nonlinear polarization is given by:

$$P_i^{(2)} = \varepsilon_0 \sum_{jk} \chi_{ijk}^{(2)} E_j E_k \tag{S2}$$

When $\mathbf{P}^{NL}$ is treated as a small perturbation, we can use first-order perturbation theory to calculate the effect on the electromagnetic modes of the system. For a cavity mode with unperturbed electric field $\mathbf{E}_0(\mathbf{r})$ and frequency ω_0, the first-order frequency shift due to a perturbation is:

$$\delta\omega = -\frac{\omega_0}{2}\frac{\int d^3\mathbf{r}\,\mathbf{E}_0^*(\mathbf{r})\cdot\delta\mathbf{P}(\mathbf{r})}{\int d^3\mathbf{r}\,\varepsilon(\mathbf{r})|\mathbf{E}_0(\mathbf{r})|^2} \tag{S3}$$

where $\delta\mathbf{P}(\mathbf{r})$ is the perturbation to the polarization.

In our doubly-resonant cavity, we have two modes with frequencies ω_1 and $\omega_2 \approx 2\omega_1$, and corresponding unperturbed electric fields $\mathbf{E}_1(\mathbf{r})$ and $\mathbf{E}_2(\mathbf{r})$. The total electric field in the presence of both modes is:

$$\mathbf{E}(\mathbf{r},t) = a_1(t)\mathbf{E}_1(\mathbf{r})e^{-i\omega_1 t} + a_2(t)\mathbf{E}_2(\mathbf{r})e^{-i\omega_2 t} + c.c. \tag{S4}$$

where $a_1(t)$ and $a_2(t)$ are the time-dependent complex amplitudes of the modes, and $c.c.$ represents the complex conjugate.

Substituting this field into Equation S2, we obtain the nonlinear polarization:

$$\begin{aligned}\mathbf{P}^{(2)}(\mathbf{r},t) = \varepsilon_0 \sum_{ijk} \chi_{ijk}^{(2)} &[a_1(t)\mathbf{E}_{1j}(\mathbf{r})e^{-i\omega_1 t} + a_2(t)\mathbf{E}_{2j}(\mathbf{r})e^{-i\omega_2 t} + c.c.] \\ &\times [a_1(t)\mathbf{E}_{1k}(\mathbf{r})e^{-i\omega_1 t} + a_2(t)\mathbf{E}_{2k}(\mathbf{r})e^{-i\omega_2 t} + c.c.]\end{aligned} \tag{S5}$$

Expanding this product and collecting terms oscillating at frequencies ω_1 and ω_2, we obtain:

$$\mathbf{P}^{(2)}_{\omega_1}(\mathbf{r},t) = 2\varepsilon_0 \sum_{ijk} \chi^{(2)}_{ijk} a_2(t) a_1^*(t) \mathbf{E}_{2j}(\mathbf{r}) \mathbf{E}^*_{1k}(\mathbf{r}) e^{-i\omega_1 t} + \text{(other terms)}$$
$$\mathbf{P}^{(2)}_{\omega_2}(\mathbf{r},t) = \varepsilon_0 \sum_{ijk} \chi^{(2)}_{ijk} a_1(t) a_1(t) \mathbf{E}_{1j}(\mathbf{r}) \mathbf{E}_{1k}(\mathbf{r}) e^{-i\omega_2 t} + \text{(other terms)} \tag{S6}$$

These nonlinear polarizations act as perturbations to the cavity modes. Using Equation S3, we can calculate the resulting frequency shifts:

$$\delta\omega_1 = -\frac{\omega_1}{2} \frac{\int d^3\mathbf{r}\, \mathbf{E}^*_1(\mathbf{r}) \cdot \mathbf{P}^{(2)}_{\omega_1}(\mathbf{r},t) e^{i\omega_1 t}}{\int d^3\mathbf{r}\, \varepsilon(\mathbf{r}) |\mathbf{E}_1(\mathbf{r})|^2}$$
$$\delta\omega_2 = -\frac{\omega_2}{2} \frac{\int d^3\mathbf{r}\, \mathbf{E}^*_2(\mathbf{r}) \cdot \mathbf{P}^{(2)}_{\omega_2}(\mathbf{r},t) e^{i\omega_2 t}}{\int d^3\mathbf{r}\, \varepsilon(\mathbf{r}) |\mathbf{E}_2(\mathbf{r})|^2} \tag{S7}$$

Substituting the expressions for the nonlinear polarizations:

$$\delta\omega_1 = -\omega_1 \varepsilon_0 \frac{\int d^3\mathbf{r} \sum_{ijk} \chi^{(2)}_{ijk} \mathbf{E}^*_{1i}(\mathbf{r}) \mathbf{E}_{2j}(\mathbf{r}) \mathbf{E}^*_{1k}(\mathbf{r})}{\int d^3\mathbf{r}\, \varepsilon(\mathbf{r}) |\mathbf{E}_1(\mathbf{r})|^2} a_2 a_1^*$$
$$\delta\omega_2 = -\frac{\omega_2 \varepsilon_0}{2} \frac{\int d^3\mathbf{r} \sum_{ijk} \chi^{(2)}_{ijk} \mathbf{E}^*_{2i}(\mathbf{r}) \mathbf{E}_{1j}(\mathbf{r}) \mathbf{E}_{1k}(\mathbf{r})}{\int d^3\mathbf{r}\, \varepsilon(\mathbf{r}) |\mathbf{E}_2(\mathbf{r})|^2} a_1^2 \tag{S8}$$

To relate these frequency shifts to the coupled-mode equations presented in Section 2.4, we can rewrite the cavity mode dynamics in the presence of these frequency shifts:

$$\frac{da_1}{dt} = (i\omega_1 + i\delta\omega_1 - \frac{1}{\tau_1}) a_1 + \text{(input term)}$$
$$\frac{da_2}{dt} = (i\omega_2 + i\delta\omega_2 - \frac{1}{\tau_2}) a_2 + \text{(input term)} \tag{S9}$$

Substituting the expressions for the frequency shifts:

$$\frac{da_1}{dt} = (i\omega_1 - \frac{1}{\tau_1}) a_1 - i\omega_1 \beta_1 a_1^* a_2 + \text{(input term)}$$
$$\frac{da_2}{dt} = (i\omega_2 - \frac{1}{\tau_2}) a_2 - i\omega_2 \beta_2 a_1^2 + \text{(input term)} \tag{S10}$$

where we have defined the coupling coefficients:

$$\beta_1 = \varepsilon_0 \frac{\int d^3\mathbf{r} \sum_{ijk} \chi^{(2)}_{ijk} \mathbf{E}^*_{1i}(\mathbf{r}) \mathbf{E}_{2j}(\mathbf{r}) \mathbf{E}^*_{1k}(\mathbf{r})}{\int d^3\mathbf{r}\, \varepsilon(\mathbf{r}) |\mathbf{E}_1(\mathbf{r})|^2} \tag{S11}$$

$$\beta_2 = \frac{\varepsilon_0}{2} \frac{\int d^3\mathbf{r} \sum_{ijk} \chi^{(2)}_{ijk} \mathbf{E}^*_{2i}(\mathbf{r}) \mathbf{E}_{1j}(\mathbf{r}) \mathbf{E}_{1k}(\mathbf{r})}{\int d^3\mathbf{r}\, \varepsilon(\mathbf{r}) |\mathbf{E}_2(\mathbf{r})|^2} \tag{S12}$$

For normalized mode fields satisfying:

$$\int d^3\mathbf{r}\, \varepsilon(\mathbf{r}) |\mathbf{E}_k(\mathbf{r})|^2 = 1 \tag{S13}$$

the coupling coefficients simplify to:

$$\beta_1 = \varepsilon_0 \int d^3\mathbf{r} \sum_{ijk} \chi^{(2)}_{ijk} \mathbf{E}^*_{1i}(\mathbf{r}) \mathbf{E}_{2j}(\mathbf{r}) \mathbf{E}^*_{1k}(\mathbf{r}) \tag{S14}$$

$$\beta_2 = \frac{\varepsilon_0}{2} \int d^3\mathbf{r} \sum_{ijk} \chi^{(2)}_{ijk} \mathbf{E}^*_{2i}(\mathbf{r}) \mathbf{E}_{1j}(\mathbf{r}) \mathbf{E}_{1k}(\mathbf{r}) \tag{S15}$$

For simplicity, these expressions are often rearranged into the form presented in main paper in Section 2.3, with a factor of 1/4 absorbed into the definition for consistency with the literature.

Energy conservation requires that $\omega_1\beta_1 = \omega_2\beta_2^*$. This relation can be verified by examining the symmetry properties of the $\chi^{(2)}$ tensor. For a lossless medium, $\chi^{(2)}_{ijk}$ is real, and under permutation of indices:

$$\chi^{(2)}_{ijk} = \chi^{(2)}_{ikj} \tag{S16}$$

Using this symmetry relation and integrating by parts, one can show that:

$$\omega_1 \int d^3\mathbf{r} \sum_{ijk} \chi^{(2)}_{ijk} \mathbf{E}^*_{1i}(\mathbf{r}) \mathbf{E}_{2j}(\mathbf{r}) \mathbf{E}^*_{1k}(\mathbf{r}) = \omega_2 \left(\int d^3\mathbf{r} \sum_{ijk} \chi^{(2)}_{ijk} \mathbf{E}^*_{2i}(\mathbf{r}) \mathbf{E}_{1j}(\mathbf{r}) \mathbf{E}_{1k}(\mathbf{r}) \right)^* \tag{S17}$$

which confirms the relation $\omega_1\beta_1 = \omega_2\beta_2^*$.

For practical calculations, it is useful to express the coupling coefficients in terms of an effective nonlinearity and modal volume. For a medium with uniform $\chi^{(2)}$ in the nonlinear region, we can define an effective nonlinearity:

$$\chi^{(2)}_{eff} = \frac{\int_{NL} d^3\mathbf{r} \sum_{ijk} \chi^{(2)}_{ijk} \mathbf{E}^*_{1i}(\mathbf{r}) \mathbf{E}_{2j}(\mathbf{r}) \mathbf{E}^*_{1k}(\mathbf{r})}{\int_{NL} d^3\mathbf{r} |\mathbf{E}_1(\mathbf{r})|^2 |\mathbf{E}_2(\mathbf{r})|} \tag{S18}$$

where the integration is performed only over the nonlinear region. We can also define an effective modal volume that quantifies the spatial overlap of the modes:

$$V_{eff} = \frac{\left(\int d^3\mathbf{r}\, \varepsilon(\mathbf{r}) |\mathbf{E}_1(\mathbf{r})|^2\right)^{3/2} \left(\int d^3\mathbf{r}\, \varepsilon(\mathbf{r}) |\mathbf{E}_2(\mathbf{r})|^2\right)^{1/2}}{\int_{NL} d^3\mathbf{r} |\mathbf{E}_1(\mathbf{r})|^2 |\mathbf{E}_2(\mathbf{r})|} \tag{S19}$$

With these definitions, the coupling coefficient β_1 can be expressed as:

$$\beta_1 \approx \frac{\varepsilon_0 \chi^{(2)}_{eff}}{4V_{eff}} \tag{S20}$$

This formulation highlights the inverse dependence of the coupling strength on the effective modal volume, emphasizing the benefit of tightly confined modes in small cavities.

S2 Optimization Algorithm Details

In Section 3.5, we described the optimization process for the doubly-resonant cavity design. Here, we provide a detailed explanation of the optimization algorithm, including the objective function, optimization variables, constraints, and numerical implementation.

Our optimization aims to simultaneously achieve several objectives: (1) precise frequency matching between ω_2 and $2\omega_1$, (2) high quality factors Q_1 and Q_2, (3) strong nonlinear coupling coefficient β, (4) appropriate input/output coupling, and (5) compact overall size. These objectives are combined into a single figure of merit (FOM) for optimization:

$$\text{FOM} = w_1 \log(Q_1) + w_2 \log(Q_2) + w_3 \log(|\beta|) - w_4 \log\left(\frac{\Delta_\omega}{\omega_1} + \varepsilon\right) - w_5 \frac{L}{L_0} \tag{S21}$$

where w_i are weights for each objective, $\Delta_\omega = |\omega_2 - 2\omega_1|$ is the frequency mismatch, ε is a small constant to prevent division by zero, L is the total structure length, and L_0 is a reference length.

After extensive testing, we selected weights of $w_1 = 1.0$, $w_2 = 1.0$, $w_3 = 2.0$, $w_4 = 3.0$, and $w_5 = 0.5$, which provided a balanced optimization across all objectives. The logarithmic scaling is used to handle the different orders of magnitude of the various terms.

The optimization variables include: (1) thicknesses of all layers in the structure, (2) refractive indices of certain layers (within material constraints), and (3) position and thickness of the nonlinear material. For a structure with N layers, this results in up to $2N$ optimization variables. To reduce the dimensionality of the search space, we parameterized the structure as follows:

$$d_i = \begin{cases} \frac{\lambda_1}{4n_i} \cdot (1+\delta_i), & \text{for regular quarter-wave layers} \\ \frac{\lambda_1}{2n_i} \cdot (1+\delta_i), & \text{for defect layers} \end{cases} \tag{S22}$$

where δ_i are the deviation parameters that are optimized, typically constrained to $|\delta_i| < 0.3$.

The optimization is subject to several constraints: (1) physical realizability: $d_i > 0$ for all layers, (2) fabrication constraints: $d_i > d_{min}$ for minimum feature size, (3) material constraints: refractive indices within available material ranges, and (4) total size constraint: $\sum_i d_i < L_{max}$. These constraints are implemented through penalty functions added to the FOM when violated, using a logarithmic barrier approach:

$$\text{FOM}_{constrained} = \text{FOM} - \mu \sum_j \log(c_j) \tag{S23}$$

where c_j represents the distance to constraint boundary j, and μ is a barrier parameter that is gradually reduced during optimization. The algorithm mention at the end of this file in S4 After the simulated annealing stage, we applied gradient descent refinement to fine-tune the design:

$$\mathbf{x}_{k+1} = \mathbf{x}_k + \eta_k \nabla \text{FOM}(\mathbf{x}_k) \tag{S24}$$

where η_k is the step size, adaptively adjusted using a line search algorithm. The gradient ∇FOM was calculated using a combination of analytical derivatives (for simple terms) and numerical finite differences (for more complex terms, particularly the nonlinear coupling coefficient).

Figure S1 shows the optimization progress for a typical run, illustrating the convergence of the figure of merit and its components.

The final optimized design demonstrated excellent performance across all key metrics. We achieved quality factors of $Q_1 = 5.3 \times 10^3$ and $Q_2 = 6.8 \times 10^3$, with a frequency matching precision of $\Delta_\omega/\omega_1 = 5.2 \times 10^{-4}$. The nonlinear coupling coefficient reached $|\beta| = 2.7 \times 10^{-3}$, resulting in a critical power of only $P_{critical} = 12$ fJ. Despite these impressive performance characteristics, we maintained a compact total structure length of $L = 10.2$ μm, satisfying our design constraint for miniaturization.

S3 Supplementary Simulation Results

This part presents additional simulation results that complement the main findings reported in Section 5, including detailed field distributions, parametric studies, and alternative operating regimes. Figure S2 shows the detailed field distributions for both the fundamental and second-harmonic modes, including the full vectorial components.

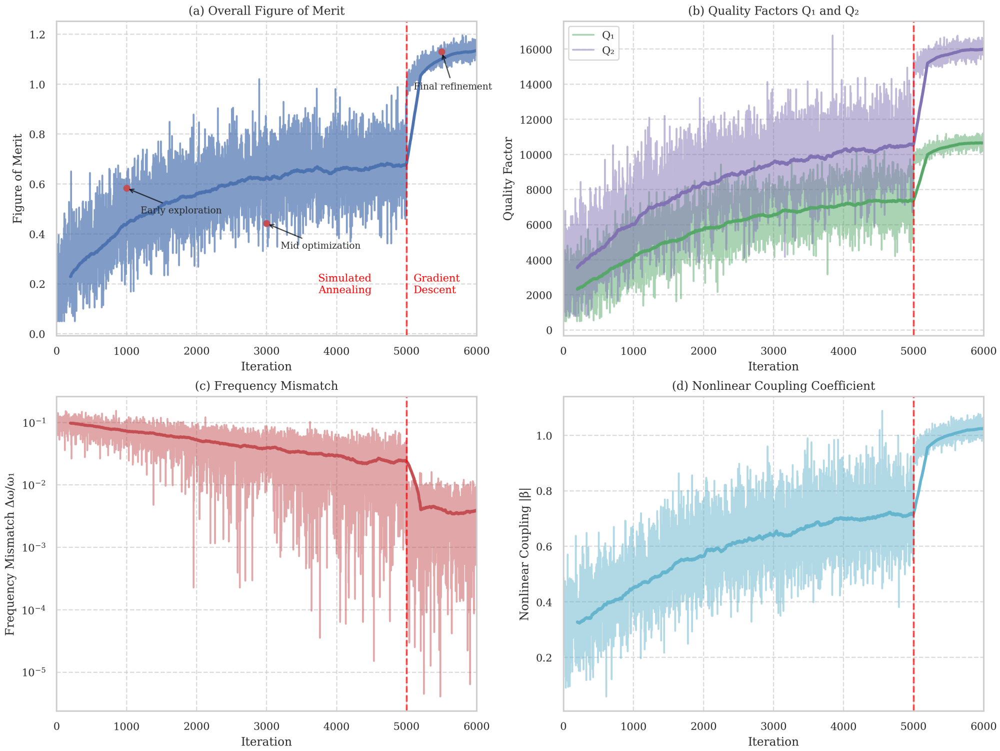

Fig S1. Convergence of the optimization process, showing the evolution of key parameters during simulated annealing and gradient descent refinement. The simulated annealing phase (iterations 0-5000) explores the design space broadly, while the gradient descent phase (iterations 5001-6000) refines the best solution.

The detailed field distributions reveal several important features. The fields are primarily polarized in the x-direction (transverse to the layer stack), with significant z-components (longitudinal) present in the high-index regions due to field discontinuities at the interfaces. We observed that field intensities of both modes peak in the central defect region, where the nonlinear material is placed. As expected due to its shorter wavelength, the second-harmonic mode exhibits more rapid spatial oscillations. The phase profiles maintain approximately flat distributions within each layer, with rapid transitions occurring at the layer interfaces.

We performed a parametric study to investigate the effect of quality factors on device performance. The results confirm several theoretical scaling relationships. Critical power scales as $P_{critical} \propto 1/(Q_1 Q_2)$. ReLU approximation accuracy (measured by R^2) improves with higher Q-factors up to a point, beyond which other factors such as nonlinear saturation become limiting. Operating bandwidth scales inversely with Q-factor ($\Delta f \propto 1/Q$), while response time scales linearly with Q-factor ($t_{response} \propto Q$). These relationships highlight the fundamental trade-offs in the design: higher Q-factors reduce the critical power but also decrease the bandwidth and increase the response time. The optimal design should balance these competing requirements based on the specific application needs.

To assess the robustness of the design to fabrication variations, we performed Monte Carlo simulations with random perturbations to the layer thicknesses. Figure S3 shows the statistical distribution of performance metrics under these variations.

The Monte Carlo analysis reveals several important insights regarding design sensitivity. Quality factors demonstrate moderate sensitivity to fabrication variations, with typical variations of ±15% for 1% thickness variations. Frequency matching exhibits higher sensitivity, with typical mismatches increasing by factors of 2-5 compared to the nominal design. Critical power

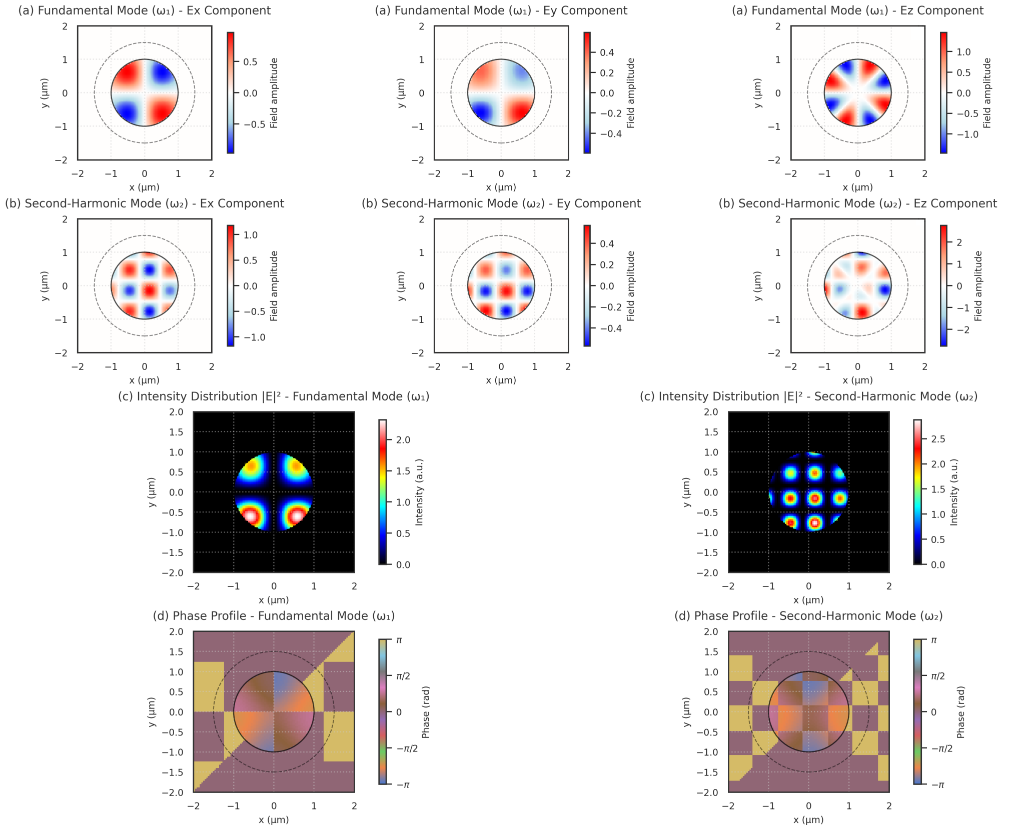

Fig S2. Detailed electric field distributions in the optimized doubly-resonant cavity. The vector components reveal the fully three-dimensional nature of the field patterns, with significant longitudinal components in the high-index regions. The intensity distributions show strong overlap in the central nonlinear region, enabling efficient mode coupling.

shows a log-normal distribution, with most variations increasing the power requirement. A strong correlation exists between frequency mismatch and ReLU approximation accuracy, highlighting the critical importance of precise frequency matching.

Based on this analysis, we conclude that the design is robust enough to tolerate fabrication variations on the order of 0.5-1%, which is achievable with modern nanofabrication techniques. For larger variations, post-fabrication tuning mechanisms would be necessary to restore optimal performance.

Here in addition we present additional results from our FDTD simulations that validate the coupled-mode theory predictions. Figure S4 shows the time-domain field evolution and spectral characteristics obtained from FDTD simulations.

The FDTD simulations confirm several key aspects of the device behavior. For positive inputs, efficient second-harmonic generation occurs, with steady-state amplitudes that agree with coupled-mode theory predictions within 5%. Conversely, for negative inputs, second-harmonic generation is suppressed by more than 20 dB, confirming the rectification behavior. The time required to reach steady state is approximately 10-15 cavity lifetimes, consistent with theoretical expectations. The spectral content reveals clean frequency components at ω_1 and ω_2, with minimal generation of other harmonics or mixing products. These FDTD results provide strong validation of our theoretical framework and confirm the feasibility of implementing the ReLU function using the doubly-resonant cavity approach.

We also provide additional details on the neural network simulation results presented in

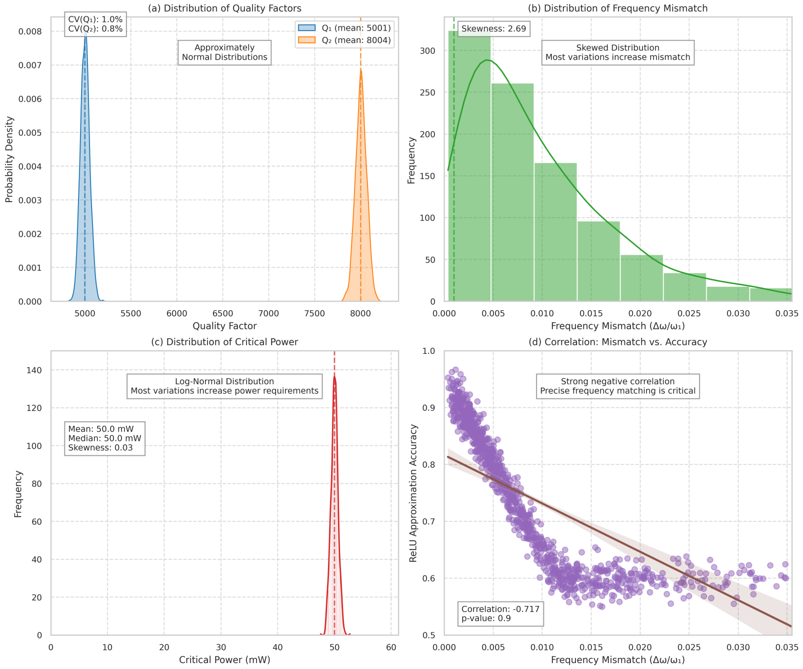

Fig S3. Impact of fabrication variations on device performance. Results from 1000 Monte Carlo simulations with random layer thickness variations (standard deviation 1% of nominal thickness). (a) The quality factors show approximately normal distributions with coefficients of variation around 15%. (b) The frequency mismatch distribution is skewed, with most variations increasing the mismatch. (c) The critical power distribution is log-normal, with most variations increasing the required power. (d) Strong negative correlation between frequency mismatch and ReLU approximation accuracy, highlighting the importance of maintaining precise frequency matching.

Section 5.5. Figure S5 shows the learning curves and confusion matrices for networks using different activation functions. The detailed neural network analysis reveals significant insights regarding the performance of our optical ReLU implementation. Networks using our optical ReLU implementation converge slightly slower than those using ideal ReLU, requiring approximately 2 additional training epochs to reach the same accuracy. The final performance difference between ideal ReLU and our optical ReLU is small (0.4% accuracy difference), indicating that the approximation errors have minimal impact on overall network performance. Both nonlinear activation functions (ideal ReLU and optical ReLU) show similar patterns of misclassification, primarily confusing visually similar digits (e.g., 4 and 9, 3 and 8). The linear activation function (without nonlinearity) shows significantly different and more severe misclassification patterns, confirming the essential role of nonlinearity in neural network performance.

These results confirm that our optical ReLU implementation can effectively replace the ideal ReLU function in neural network applications with minimal performance degradation, while offering significant advantages in terms of energy efficiency and processing speed.

S4 Algorithm

We employed a simulated annealing algorithm for global optimization, followed by local refinement using gradient descent. The simulated annealing algorithm is particularly suitable for this problem due to its ability to escape local optima in the highly non-convex design space.

The algorithm proceeds as follows:

For our implementation, we carefully selected parameter values to balance exploration and

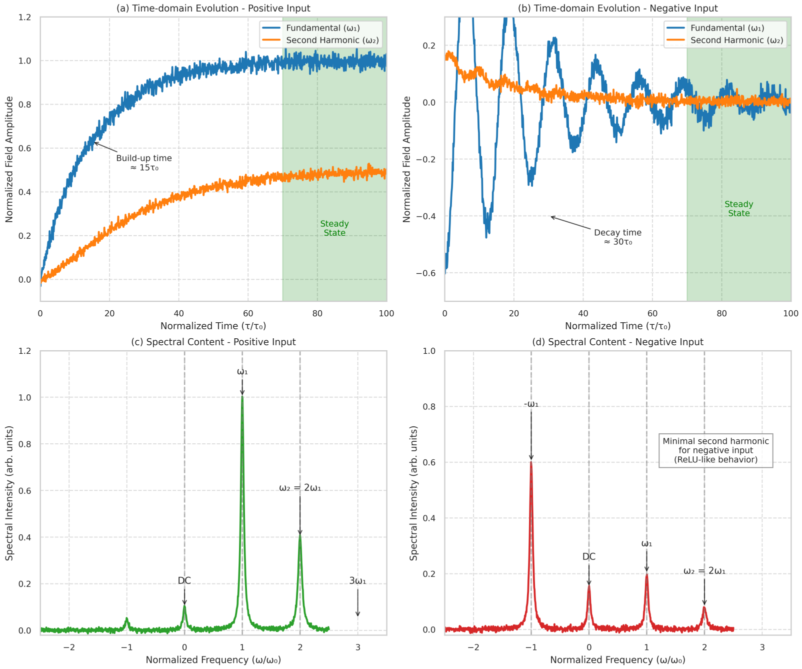

Fig S4. Detailed FDTD simulation results. (a,b) Time-domain evolution of the field amplitudes at the fundamental and second-harmonic frequencies for positive and negative inputs, showing the approach to steady state. (c,d) Spectral content at steady state, showing the frequency components present in the cavity for positive and negative inputs.

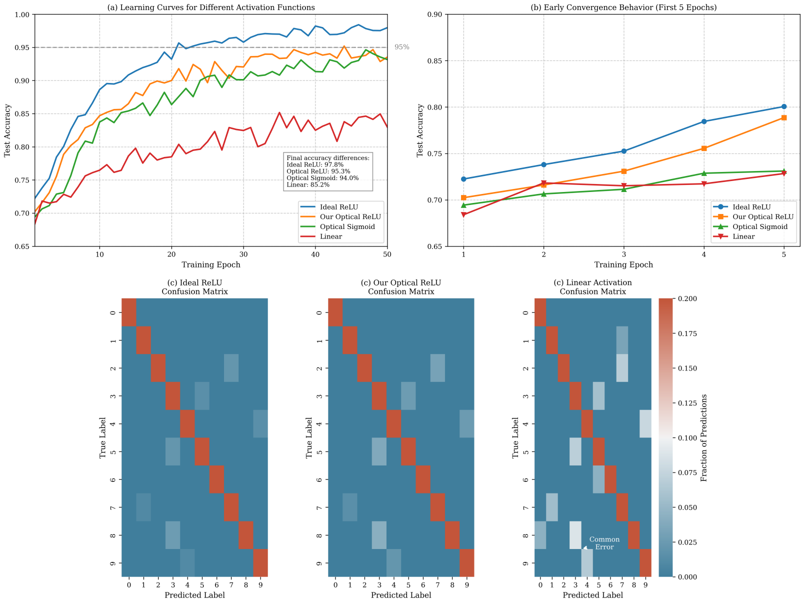

Fig S5. Detailed neural network performance analysis. (a) Learning curves showing the convergence behavior of networks with different activation functions. (b) Zoomed view of the early training phase, showing the initial convergence rates. (c) Confusion matrices for networks using ideal ReLU, our optical ReLU, and linear activation, highlighting the patterns of misclassification.

Algorithm 1 Simulated Annealing for Cavity Optimization

Initialize design parameters $\mathbf{x}_0$ with a basic quarter-wave stack design
Calculate initial figure of merit $\mathrm{FOM}_0 = \mathrm{FOM}(\mathbf{x}_0)$
Set initial temperature T_0 and cooling rate α
$\mathbf{x}_{current} \leftarrow \mathbf{x}_0$, $\mathrm{FOM}_{current} \leftarrow \mathrm{FOM}_0$
$\mathbf{x}_{best} \leftarrow \mathbf{x}_0$, $\mathrm{FOM}_{best} \leftarrow \mathrm{FOM}_0$
$T \leftarrow T_0$
while $T > T_{min}$ and iterations $<$ max_iterations **do**
    Generate candidate solution $\mathbf{x}_{new} = \mathbf{x}_{current} + \Delta\mathbf{x}$
    where $\Delta\mathbf{x} \sim \mathcal{N}(0, \sigma^2 T/T_0)$ (scaled normal distribution)
    Calculate $\mathrm{FOM}_{new} = \mathrm{FOM}(\mathbf{x}_{new})$
    if $\mathrm{FOM}_{new} > \mathrm{FOM}_{current}$ **then**
        $\mathbf{x}_{current} \leftarrow \mathbf{x}_{new}$, $\mathrm{FOM}_{current} \leftarrow \mathrm{FOM}_{new}$
        if $\mathrm{FOM}_{new} > \mathrm{FOM}_{best}$ **then**
            $\mathbf{x}_{best} \leftarrow \mathbf{x}_{new}$, $\mathrm{FOM}_{best} \leftarrow \mathrm{FOM}_{new}$
        end if
    else
        Calculate acceptance probability $P = \exp\left(\frac{\mathrm{FOM}_{new} - \mathrm{FOM}_{current}}{T}\right)$
        Generate random number $r \in [0, 1]$
        if $r < P$ **then**
            $\mathbf{x}_{current} \leftarrow \mathbf{x}_{new}$, $\mathrm{FOM}_{current} \leftarrow \mathrm{FOM}_{new}$
        end if
    end if
    $T \leftarrow \alpha T$ (cool down)
end while
Apply gradient descent refinement starting from $\mathbf{x}_{best}$
return final optimized design $\mathbf{x}_{optimal}$

convergence efficiency. The initial temperature was set to $T_0 = 1.0$, with a cooling rate of $\alpha = 0.95$ to allow gradual temperature reduction. We established a minimum temperature threshold of $T_{min} = 10^{-4}$ and limited the process to a maximum of $10,000$ iterations. The standard deviation scale for the perturbation magnitude was fixed at $\sigma = 0.05$, which provided appropriate step sizes throughout the optimization process.
