# Supplementary material for: All-optical doubly resonant cavities for energy-efficient ReLU function in nanophotonic deep learning
Source: PLoS One. 2026 Jun 17;21(6):e0345850. doi: 10.1371/journal.pone.0345850 (PMC13274909; doi:10.1371/journal.pone.0345850)
Supplement: Attachment — Submitted filename: Response to Reviewers.pdf [file pone.0345850.s003.pdf]

Response to Reviewers

Manuscript: "All-Optical Doubly Resonant Cavities for Energy-Efficient ReLU Function in Nanophotonic Deep Learning"

Authors: Amirreza Ahmadnejad, Mohammad Mehrdad Asadi, Somayyeh Koohi

Dear Editor and Reviewers,

We sincerely thank the reviewers for their thorough evaluation and constructive feedback on our manuscript. We have carefully addressed all the comments and believe that the revisions have significantly strengthened the paper. Below, we provide detailed point-by-point responses to each reviewer's comments. All major changes in the revised manuscript are highlighted in yellow for easy identification.

Response to Reviewer #1

We thank Reviewer #1 for the positive evaluation and thoughtful suggestions that have helped us improve the manuscript's comprehensiveness and clarity.

Comment 1a:

The ReLU implementation relies on encoding the sign of the input in the optical phase. This is elegant but may not align with typical signal encoding in ONNs.

Response: We appreciate this important observation regarding the compatibility of our phase-encoding scheme with existing optical neural network architectures. We have now added a comprehensive discussion addressing this concern.

Changes made: We have added a new subsection in **Section 2 (Theoretical Framework)** titled **"Compatibility with Optical Neural Network Architectures"** . This addition includes:

- A detailed explanation of how our phase-encoding scheme ($\phi_1 = 0$ for positive inputs, $\phi_1 = \pi$ for negative inputs) naturally aligns with coherent optical neural network architectures, particularly Mach-Zehnder interferometer (MZI) meshes [6, 3].
- Clarification that in MZI-based systems, the output fields inherently carry both amplitude and phase information, with constructive and destructive interference naturally generating phase relationships of 0 or π depending on the sign of matrix elements. Therefore, the transition from MZI-based linear layers to our phase-sensitive ReLU is seamless, requiring no additional phase encoding circuitry.
- Discussion of similar phase-based encoding schemes successfully employed in other compact nanophotonic computing architectures [5, 1], demonstrating the practicality of phase-dependent optical operations.
- For integration with incoherent or intensity-based approaches, we note that modern integrated electro-optic phase modulators in thin-film lithium niobate can provide the required phase encoding with minimal overhead (operating at speeds exceeding 100 GHz with insertion losses below 1 dB [8]).

This comprehensive addition positions our phase-encoding approach within the broader context of optical neural network architectures and demonstrates its natural compatibility with the most widely studied and scalable implementations. The added text is highlighted in yellow in the revised manuscript.

Comments 1b and 1c (addressed together):

(b) The comparison could be expanded to include more recent nanophotonic nonlinear activation approaches.

(c) A quantitative benchmarking table summarizing energy, footprint, and speed across methods would be valuable.

Response: We agree that a more comprehensive comparison with recent approaches would significantly enhance the manuscript. We have substantially expanded our comparison to provide a thorough survey of the field.

Changes made: We have replaced the original **Table 5** with a new, comprehensive **Table 6** titled **"Comprehensive comparison of optical nonlinear activation function implementations for neural networks"** in **Section 4**. The key improvements include:

- **Expanded coverage:** The new table includes 22 implementations (compared to 5 in the original), spanning from 2013 to 2023, providing a comprehensive historical perspective.
- **Systematic categorization:** Implementations are organized by physical mechanism into six categories:
  1. $\chi^{(2)}$ nonlinear processes (including our work and PPLN waveguides)
  2. $\chi^{(3)}$ nonlinear processes (microring resonators, photonic accelerators)
  3. Saturable absorption (graphene, 2D materials, ENZ materials)
  4. Phase-change materials (GST-based implementations)
  5. Hybrid/interference-based approaches (optical scattering units, coherent circuits)
  6. Neuromorphic/laser-based neurons (VCSELs, reservoir computing)
- **Quantitative metrics:** Each entry includes energy per activation, response time, device size, function type, and platform/material, directly addressing the request for comprehensive benchmarking.
- **Extended discussion:** We have added approximately 500 words of detailed analysis following the table, systematically comparing our approach with each category and highlighting the trade-offs between energy efficiency, speed, footprint, and functionality. This discussion emphasizes how our doubly-resonant cavity uniquely combines femtojoule-level energy efficiency (12 fJ), sub-picosecond response time (400 fs), and micrometer-scale footprint (10 μm) while maintaining versatility to implement multiple activation functions (ReLU, ELU, GELU).

The new table and accompanying text are highlighted in yellow in the revised manuscript. We believe this comprehensive comparison clearly positions our work within the state-of-the-art and demonstrates its unique advantages.

Comment 1d:

The paper mentions classification accuracy within 0.4% of electronic ReLU, but details are not given.

Response: We apologize for the insufficient detail in our original presentation of the neural network performance. We have now provided a comprehensive quantitative analysis.

Changes made: We have substantially expanded the discussion in **Section 4.3** with a new subsection titled **"Detailed classification performance analysis."** The additions include:

- **Specific accuracy metrics:** Test accuracy of 97.1% (optical ReLU) vs. 97.3% (ideal electronic ReLU), corresponding to a difference of 0.2% or approximately 10 additional misclassifications among 5,000 test images.
- **Training dynamics:** The network with optical ReLU required 12 epochs to converge compared to 10 epochs for ideal ReLU, with final training losses of 2.4×10^{-2} and 2.1×10^{-2}, respectively.
- **Error pattern analysis:** The confusion matrices (Figures 9d,e) show identical error patterns between implementations, with highest confusion between visually similar digit pairs, indicating that the performance gap is due to minor activation function deviations rather than fundamental limitations.
- **Detailed error analysis:** We quantified the mean squared error ($\text{MSE} = 8.1 \times 10^{-3}$) between our experimental optical ReLU and the ideal function. The primary deviations occur in: (1) sub-threshold region with 3% residual output for small negative inputs (contributing 0.15% accuracy loss), and (2) slight saturation effects (5% deviation) for large positive inputs above 25 fJ (contributing 0.05% accuracy loss).
- **Ablation studies:** We performed numerical experiments isolating each deviation source, confirming that sub-threshold leakage is the dominant factor in the 0.2% accuracy difference.
- **Noise robustness:** The network maintains ¿95% accuracy for signal-to-noise ratios above 20 dB, demonstrating practical robustness to optical noise.
- **Comparative performance:** We contextualize our results within the broader field, noting that this small performance gap is well within acceptable limits and comparable to other optical implementations while offering superior energy efficiency and compactness.

This expanded analysis provides the quantitative detail requested by the reviewer and demonstrates that our optical implementation achieves near-ideal performance. The additions are highlighted in yellow in the revised manuscript.

Response to Reviewer #2

We thank Reviewer #2 for the careful reading and specific technical suggestions that have helped us improve the manuscript's technical completeness and clarity.

Comment 2a:

The cavity parameters should be described in greater detail, including the lattice period and refractive index distribution. The stated cavity dimension of approximately 10 μm alone does not sufficiently convey the complete structural configuration of the cavity.

Response: We agree that the original manuscript lacked sufficient structural detail for complete understanding and reproducibility. We have now provided comprehensive cavity parameters.

Changes made: We have added extensive structural details in **Section 3.1 (Linear Cavity Design)** (around line 310, following the discussion of quarter-wave stacks). The additions include:

- **New comprehensive table:** We have added **Table 3 (or renumbered accordingly)** titled **"Complete structural parameters of the doubly-resonant cavity,"** which provides a systematic breakdown of all cavity parameters organized into five categories:
    1. Material properties: Refractive indices ($n_H = 3.40$, $n_L = 1.00$), nonlinear susceptibility ($\chi^{(2)} = 100$ pm/V, $d_{\text{eff}} = 50$ pm/V)
    2. Quarter-wave stack (DBR mirrors): Layer thicknesses (d_H, d_L), lattice period ($\Lambda = 502$ nm), number of layer pairs ($N_L = 7$), total mirror length ($L_{DBR} = 3.51\mu$m)
    3. Defect region: Modified layer thicknesses ($d_{H'}$, $d_{H''}$, $d_{L,\text{defect}}$), defect structure (H'-L-H"-L-H'), total defect length ($L_{\text{defect}} = 1.23\mu$m)
    4. Transverse dimensions: Ridge width ($w = 1.8\mu$m), film thickness ($h_{\text{total}} = 700$ nm), etch depth ($h_{\text{ridge}} = 400$ nm), effective mode area ($A_{\text{eff}} = 2.1\mu$ m^2)
    5. Overall cavity: Total length ($L_{\text{total}} = 8.25\mu$ m), complete footprint (10×2 μm^2), design wavelengths
- **Complete refractive index distribution:** We explicitly state the piecewise constant profile: $n(z) = n_H = 3.40$ for AlGaAs layers and $n(z) = n_L = 1.00$ for air gaps, with the spatial arrangement defined by the lattice period and defect structure.
- **Lattice period explicitly stated:** $\Lambda = d_H + d_L = 502$ nm, which determines the periodicity of the photonic crystal structure.

These additions provide complete structural information sufficient for reproduction and detailed understanding of the cavity design. All additions are highlighted in yellow in the revised manuscript.

Comment 2b:

Including the dispersion characteristics of the cavity would improve the clarity and readability of the manuscript.

Response: We thank the reviewer for this valuable suggestion. Dispersion characteristics are indeed crucial for understanding the frequency matching precision and operational bandwidth of our device. We have now included a comprehensive treatment of dispersion.

Changes made: We have added a new subsection in **Section 3.1 (Linear Cavity Design)** (immediately following the structural parameters discussion) titled **"Dispersion characteristics and frequency matching."** The additions include:

- **Material dispersion:** Complete Sellmeier equation for $Al_{0.3}Ga_{0.7}As$:

$$n^2(\lambda) = A + \frac{B\lambda^2}{\lambda^2 - C^2}$$

with parameters $A = 10.906$, $B = 0.97501\mu m^2$, $C = 0.52886\mu\text{m}$ [2]. We provide explicit values: $n(\lambda_1 = 1550 \text{ nm}) = 3.40$ and $n(\lambda_2 = 775 \text{ nm}) = 3.52$.

- **Material dispersion parameters:** Wavelength derivatives $\mathrm{d}n/\mathrm{d}\lambda \approx -0.015\ \mu m^{-1}$ at λ_1 and $-0.048\mu\text{m}^{-1}$ at λ_2.

- **Group index:** Calculated values $n_{g,1} \approx 3.42$ at the fundamental and $n_{g,2} \approx 3.56$ at the second harmonic, which determine pulse propagation velocities.

- **Group velocity dispersion (GVD):** Quantified as $\beta_{2,1} \approx 180$ fs²/mm at the fundamental wavelength and $\beta_{2,2} \approx 420$ fs²/mm at the second harmonic, demonstrating minimal pulse broadening for femtosecond pulses.

- **Cavity dispersion - Free spectral range:** Calculated FSR $= c/(2n_{\text{eff}}L_{\text{eff}}) \approx 7.6$ THz, showing the frequency spacing between adjacent cavity modes and ensuring isolation of the fundamental and second-harmonic resonances.

- **Frequency matching precision:** Explicit statement that $|\omega_2 - 2\omega_1|/\omega_1 < 5.2 \times 10^{-4}$, which is well within the cavity linewidths ($\Delta\omega_1/\omega_1 = 1.9 \times 10^{-4}$ and $\Delta\omega_2/\omega_2 = 1.5 \times 10^{-4}$).

- **Acceptance bandwidth:** Resonance linewidth $\Delta\lambda_1 \approx 0.29$ nm (corresponding to 36 GHz bandwidth), determined by the cavity quality factor rather than phase-matching considerations.

- **Temperature tuning:** Thermo-optic coefficient $\mathrm{d}n/\mathrm{d}T \approx 2 \times 10^{-4}\ \text{K}^{-1}$ resulting in frequency shift $\mathrm{d}\omega_1/\mathrm{d}T \approx -11$ GHz/K, enabling fine-tuning over a few degrees Celsius.

- **Comparison with phase-matched approaches:** Discussion of how our resonant cavity approach offers advantages in dispersion management compared to conventional periodically-poled waveguides, with relaxed fabrication tolerances while maintaining comparable bandwidth.

This comprehensive addition provides complete characterization of the dispersion properties and their impact on device performance. We have also added relevant numerical values to the structural parameters table (if space permits). All additions are highlighted in yellow in the revised manuscript.

Comment 2c:

It should be explicitly clarified whether a two-dimensional (2D) or three-dimensional (3D) finite-difference time-domain (FDTD) simulation method has been employed.

Response: We apologize for this omission in the original manuscript. We have now explicitly clarified the dimensionality of our simulations and justified the approach.

Changes made: We have added explicit clarification at the **beginning of Section 4 (Numerical Simulation Methods)** (immediately after the section title, around line 451). The additions include:

- **Explicit statement:** A new subsection titled **"Two-dimensional FDTD simulation approach"** clearly states that all full-wave electromagnetic simulations were performed using two-dimensional (2D) FDTD methods implemented in Lumerical FDTD Solutions.

- **Justification of 2D approach:** We explain that the 2D approximation is justified by the translational invariance of our cavity structure along the transverse direction perpendicular to both the propagation axis and the direction of refractive index variation. We simulate in the x-z plane (where z is the propagation direction and x is the direction of index modulation), assuming no field variation in the y-direction.

- **Treatment of transverse confinement:** We clarify that transverse mode confinement effects in the ridge waveguide are accounted for through an effective index approximation. The effective refractive index ($n_{\text{eff}} \approx 2.4$) is calculated using a separate 2D mode solver for the waveguide cross-section (width $w = 1.8\mu$m, ridge height $h_{\text{ridge}} = 400$ nm) and then used in the 2D FDTD simulations along the cavity axis.

- **Validation of approach:** We reference established literature demonstrating the validity of this approach for photonic crystal cavities and DBR structures where longitudinal dynamics dominate [4, 7].

- **Consistency throughout:** We have also reviewed Section 4 and added the "2D" qualifier where appropriate (e.g., "2D FDTD simulation domain," "2D full-wave simulations") to maintain consistency.

This addition explicitly clarifies the simulation dimensionality, justifies the approach, and demonstrates its validity for our cavity geometry. The additions are highlighted in yellow in the revised manuscript.

References

[1] Johannes Feldmann, Nathan Youngblood, Maxim Karpov, Helge Gehring, Xuan Li, Manon Stappers, Manuel Le Gallo, Xiao Fu, Anton Lukashchuk, Arslan S Raja, et al. Parallel convolutional processing using an integrated photonic tensor core. *Nature*, 589(7840):52–58, 2021.

[2] S Gehrsitz, FK Reinhart, C Gourgon, N Herres, A Vonlanthen, and H Sigg. The refractive index of al$_x$ga$_{1-x}$as below the band gap: accurate determination and empirical modeling. *Journal of Applied Physics*, 87(11):7825–7837, 2000.

[3] Nicholas C Harris, Jacques Carolan, Darius Bunandar, Mihika Prabhu, Michael Hochberg, Tom Baehr-Jones, Michael L Fanto, A Matthew Smith, Christopher C Tison, Paul M Alsing, et al. Linear programmable nanophotonic processors. *Optica*, 5(12):1623–1631, 2018.

[4] Steven G Johnson and John D Joannopoulos. Block-iterative frequency-domain methods for maxwell's equations in a planewave basis. *Optics Express*, 8(3):173–190, 2001.

[5] Yurui Qu, Huanzheng Zhu, Yichen Shen, Jin Zhang, Chenning Tao, Pintu Ghosh, and Min Qiu. Inverse design of an integrated-nanophotonics optical neural network. *Science Bulletin*, 65(14):1177–1183, 2020.

[6] Y. Shen, N. C. Harris, S. Skirlo, M. Prabhu, T. Baehr-Jones, M. Hochberg, X. Sun, S. Zhao, H. Larochelle, D. Englund, and M. Soljačić. Deep learning with coherent nanophotonic circuits. *Nature Photonics*, 11(7):441, 2017.

[7] Allen Taflove and Susan C Hagness. *Computational electrodynamics: the finite-difference time-domain method*. Artech house, 2005.

[8] Cheng Wang, Mian Zhang, Xi Chen, Maxime Bertrand, Amirhassan Shams-Ansari, Sethumadhavan Chandrasekhar, Peter Winzer, and Marko Loncar. Integrated lithium niobate electro-optic modulators operating at cmos-compatible voltages. *Nature*, 562(7725):101–104, 2018.
